# Constructing, conducting and interpreting animal social network analysis

**DOI:** 10.1111/1365-2656.12418

**Published:** 2015-08-11

**Authors:** Damien R. Farine, Hal Whitehead

**Affiliations:** ^1^ Department of ZoologyEdward Grey Institute of Field OrnithologyUniversity of OxfordSouth Parks RoadOxfordOX1 3PSUK; ^2^ Department of Anthropology (Evolutionary)University of California Davis1 Shields AvenueDavisCA95616USA; ^3^ Smithsonian Tropical Research InstituteAnconPanama; ^4^ Department of BiologyDalhousie University1355 Oxford StHalifaxNSCanadaB3H 4J1

**Keywords:** fission‐fusion dynamics, group living, methods, social behaviour, social dynamics, social network analysis, social organisation

## Abstract

1. Animal social networks are descriptions of social structure which, aside from their intrinsic interest for understanding sociality, can have significant bearing across many fields of biology.
2. Network analysis provides a flexible toolbox for testing a broad range of hypotheses, and for describing the social system of species or populations in a quantitative and comparable manner. However, it requires careful consideration of underlying assumptions, in particular differentiating real from observed networks and controlling for inherent biases that are common in social data.
3. We provide a practical guide for using this framework to analyse animal social systems and test hypotheses. First, we discuss key considerations when defining nodes and edges, and when designing methods for collecting data. We discuss different approaches for inferring social networks from these data and displaying them. We then provide an overview of methods for quantifying properties of nodes and networks, as well as for testing hypotheses concerning network structure and network processes. Finally, we provide information about assessing the power and accuracy of an observed network.
4. Alongside this manuscript, we provide appendices containing background information on common programming routines and worked examples of how to perform network analysis using the *r* programming language.
5. We conclude by discussing some of the major current challenges in social network analysis and interesting future directions. In particular, we highlight the under‐exploited potential of experimental manipulations on social networks to address research questions.

## Introduction

Social network analysis is a commonly used toolbox for biologists investigating the causes and consequences of complex social and ecological interactions in animal communities. It is a key technique in sociology (Wasserman & Faust 1994), where it originated in the 1930s, to investigate the link between local patterns of human relationships and social processes, such as the impact of social groups on the likelihood of being obese (Christakis & Fowler 2007). Social network analysis provides a flexible framework for analysing association or interaction data to address a broad set of biological questions (Croft, James & Krause 2008). Most fundamentally, it provides a description of social structure. Network data also forms the substrate for a range of analyses including affiliation or avoidance between conspecifics (Lusseau 2003; Croft, Krause & James 2004), interspecific interactions (Farine, Garroway & Sheldon 2012), mating behaviour (McDonald *et al*. 2013), genetic networks (Gardner *et al*. 2003) and community‐level linkages (Montoya, Pimm & Sole 2006; Olesen *et al*. 2008). The strength of social network analysis is that it provides an understanding of how local processes drive group‐level properties by taking into account the different social environments experienced by each individual (Strandburg‐Peshkin *et al*. 2013), how individual variation in social behaviour can drive population structure (Aplin *et al*. 2013; Jacoby *et al*. 2014; Snijders *et al*. 2014) and how socially transmitted quantities, such as information or disease, flow through individuals in a population (Boogert *et al*. 2008; Hamede *et al*. 2009; Kendal *et al*. 2010). The properties of individuals captured by social network analysis can then be linked to fitness (McDonald 2007; Silk *et al*. 2010; Formica *et al*. 2012; Wey *et al*. 2013; Farine & Sheldon 2015), thus framing sociality in an evolutionary context. Because it provides a means of linking social behaviour across all levels of organization, network analysis is increasingly central to many fields of biology and is quickly becoming the most commonly used approach for describing the structure of social relationships in a broad range of taxa.

**Figure 1:**
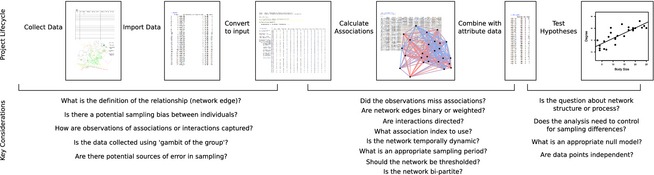
Primary steps and key considerations in the collection and analysis of animal social networks (see also Table 1).

In this paper, we focus on the application of social network analysis to non‐human animal data (see Fig. 1 & Table 1). Sections Overview of Network Analysis through Data collection methods outline the motivations for performing network analyses and give advice on defining what the network represents and collecting data. Sections Inferring associations from data and Displaying social structures provide the information required to decide how to infer edges, calculate the value of each edge and display the resulting network. Section Interpreting network metrics introduces different network metrics and what to consider when interpreting their values and distributions. Section Constructing null models explains how null models and permutation tests can be used to conduct robust statistical testing. Section Hypothesis testing in animal social networks details a number of current and future approaches for hypothesis testing in animal social network analysis. Section Estimating power and precision provides guidelines for estimating the power and precision of an observed network and background on different sources of bias. Section Remaining challenges and future directions discusses future directions that we believe will provide new insight into animal social networks. This paper is accompanied by appendices containing worked empirical and simulation examples using *R*. Because animal social network analysis has become a broad field of research, interpretation of network data is dependent on the definitions and assumptions used in each individual case. Our aim is to introduce the process of studying social behaviour using social network analysis by providing a synthesis of the approaches and considerations that are common to most studies (see Box 1). This topic is considered at greater length in the books by Croft, James & Krause (2008) and Whitehead (2008), but there have been recent advances, especially in the automated collection of very large data sets and in the development of analytical tools to tackle the novel challenges associated with generating networks from tracking data. The application of social network analysis across different domains of animal biology is discussed with greater detail in the chapters of the book edited by Krause *et al*. (2015).

Box 1 **Key Points**

- Match data collection, and node and edge definitions to the biological questions.
- Determine whether edges will be weighted or binary, and directed or undirected.
- Calculate significance by comparing observed results to a distribution of test statistics generated using permutation tests.
- Avoid thresholding the network.
- Ensure that the scale of data collection appropriately captures the biological process.
- Use appropriate sampling period for each analysis
- Maximize the number of observations per individual (and consider the potential trade‐off between sampling rate and number of individuals in the study).
- Avoid using network‐level metrics unless a high proportion of individuals in the population are identified.
- Consider and control for potential confounding effects generated by other social processes and the method of data collection.
- Keep individuals in the same order in all data used for analysis (attribute data are ordered the same as the rows/columns in the adjacency matrix).

**Table 1:** Overview of key considerations in each step of network analysis (see also Fig. 1). In addition to the key references, both Whitehead (2008) and Croft, James & Krause (2008) cover these topics in detail

Step	Important consideration	Key references
Collecting data	1. What is being observed? 1a. Associations/Gambit of the group? 1b. Interactions between individuals? 2. What data sampling method to use: focal follows, group follows, all‐event? 3. Is there observation error or bias?	General methods: Whitehead (1997), Whitehead & Dufault (1999), James, Croft & Krause (2009). Sampling methods: Altmann (1974) Gambit of the group: Franks, Ruxton & James (2010)
Building the network	1. What is the biological definition of an edge in the network? 2. What is the sampling period? 3. Is there any observation bias that can be controlled with the association index? 4. Are edges directed or undirected?	Edge inference: Psorakis *et al*. (2012). Association indices: Cairns & Schwager (1987). Affiliation indices (Whitehead & James 2015) Dynamic networks: Blonder *et al*. (2012), Hobson, Avery & Wright (2013),Pinter‐Wollman *et al*. (2013).
Hypothesis testing	1. What is the question? 2. What is the null hypothesis? 3. Is it potentially true? 4. What needs to be controlled for? 5. What test statistic(s) should be used? 6. Does the network need to be compared to randomized networks? 6a. What type of randomization is required? 7. Present effect size statistics.	General considerations: Croft *et al*. (2011b). Randomizations: Manly (1997), Bejder, Fletcher & Brager (1998), Whitehead (1999) Whitehead, Bejder & Ottensmeyer (2005), Sundaresan, Fischhoff & Dushoff (2009).

## Overview of Network Analysis

### What is a network?

The term ‘network’ can mean different things to different people. It can refer to the ‘real’ set of interactions between animals that integrate to form community dynamics. Hinde (1976) defined social structure in terms of the nature, quality and patterning of the relationships among its members, where a relationship between two individuals integrates the content, quality and patterning of their interactions. For example, animals might have genetic, affiliative, agonistic, cooperative, dominant and many other types of relationships that combine to form the ‘true’ social system (Barrat *et al*. 2004). We can call this the *real* network.

Most commonly, the networks that biologists create are analytical representations of a combined set (or subset) of measures of the true relationships. We can call these the *observed* networks. To make a simple analogy, consider that for many decades, biologists could only infer genetic structure from observed parent–offspring relationships. Researchers could only obtain the true underlying genetic relationships of a population (or pedigree which is a type of network), once DNA paternity testing was developed.

In this paper, we generally use *network* as a description for the *observed patterns of associations or interactions*. We use *network analysis* as the framework of analytical approaches that use observed networks to try and describe and visualize the real network, as well as to test hypotheses about biological phenomena related to social behaviour. For a broader perspective on this question, we point readers to Borgatti & Halgin (2011).

### Network structure

Networks consist of nodes connected by edges. *Nodes* can represent individuals, groups, classes of individuals or other entities. Each node can possess attributes, such as the identity and phenotypic traits of the individual it represents. *Edges* generally represent how two nodes relate to one another, and can be used to describe how frequently they associate or interact, or to describe other relationships (such as genetic relatedness, see Section Data collection methods). They often have numeric values (*weighted edges*), describing the strength of the relationship (e.g. rates or numbers of interactions), although it can sometimes be useful to conceive of edges as *binary* (either 0 or 1) indicating the presence or absence of a relationship (e.g. whether a male–female pair have copulated or not, McDonald *et al*. 2013). Nodes and/or edges may also vary temporally, allowing network structure to change dynamically over time (these are termed *dynamic networks*, see Section Inferring associations from data).

### Why quantify networks?

Early applications of social network methods, such as those in dolphins (Lusseau 2003) and guppies (Croft, Krause & James 2004), were largely descriptive. These often investigated variation in social roles (Lusseau & Newman 2004) and phenotypic structure in populations (Croft *et al*. 2005). More recently, the power of the network approach has been clearly demonstrated by two types of studies: those describing potential fitness consequences related to network position, and those investigating the spread of information and disease through animal networks. Network studies can thus be placed into four broad categories: (i) description of social structures (e.g. Connor, Heithaus & Barre 2001; Wittemyer, Douglas‐Hamilton & Getz 2005); (ii) studies of the causes and consequences of individual variation in network position – where ‘network position’ refers to the structural properties that arise as a consequence of an individual's phenotype or patterns of sociality (e.g. McDonald 2007; Pike *et al*. 2008; Oh & Badyaev 2010; Aplin *et al*. 2013); (iii) studies of social processes and the implications of network structure for dynamics of information (e.g. Boogert *et al*. 2008; Aplin *et al*. 2012, 2015; Allen *et al*. 2013) and disease or parasite spread across networks (e.g. Godfrey *et al*. 2009; Hamede *et al*. 2009; Fenner, Godfrey & Bull 2011; Bull, Godfrey & Gordon 2012; MacIntosh *et al*. 2012; Brockmann & Helbing 2013; VanderWaal *et al*. 2013a,b); and (iv) relationship between environment and network structure (Edenbrow *et al*. 2011; Webster *et al*. 2013; Firth & Sheldon 2015; Pinter‐Wollman 2015) and vice‐versa (Oh & Badyaev 2010; Formica *et al*. 2011; Shizuka & McDonald 2012; Farine & Sheldon 2015). The first category of studies deals with visualizing and describing the natural world (network diagrams and statistics are more easily assimilated by the human brain than raw matrices of association measures). The second category deals with individual traits and provides a framework for quantifying individual position within a broader social structure. The third focuses on the interplay between social dynamics, interaction patterns and socially mediated flows, where networks provide a unique ability to integrate the dyadic nature and non‐uniform structure of social relationships when modelling social processes. The last focuses on the external factors that shape network structure, how network structure can alter individuals’ environments and to what extent individuals can shape aspects of their environment.

Network approaches also have the potential to characterize emergent properties of social organization. A classic paper by Granovetter (1973) highlighted the potential importance of weak ties (connections that are rarely made) for connecting otherwise disparate groups or communities, in turn shaping their higher‐level structure. The idea that societies can have properties that are a function of their overall structure (i.e. emergent properties), rather than of individuals themselves, has stimulated a substantial body of theoretical research into the link between network structure and social processes, in particular the spread of disease or information (e.g. Newman 2002; Keeling 2005; Shirley & Rushton 2005; May 2006; Bansal, Grenfell & Meyers 2007; Perkins *et al*. 2009; Volz *et al*. 2011; Hock & Fefferman 2012; Whitehead & Lusseau 2012; Ashby & Gupta 2013). Many of these studies used analytical mathematical models, and this approach that has made a large contribution to our understanding of the emergent properties of networks.

Network analysis provides a common framework for studying emergent processes in animal societies. For example, many animal populations exhibit fission–fusion dynamics, which can emerge under a range of ecological pressures (Aureli *et al*. 2008). In such populations, the patterns of associations can often appear random. However, social network analysis can elucidate whether patterns of connections between phenotypes (i.e. assortment in the social network) are non‐random (Farine *et al*. 2015b). Because such processes may play a role in shaping how selection drives the evolution of social species, future efforts will benefit from a focus on the following: (i) the process of self‐organization and orderliness in social networks, (ii) what interactions (including affiliative, genetic, mating and spatial networks) underlie dynamic processes in social networks (such as transmission properties) and (iii) whether network structure can evolve in response to selection pressure (both ecological and social).

## Defining network edges

### Defining relationships (edges)

**Figure 2:**
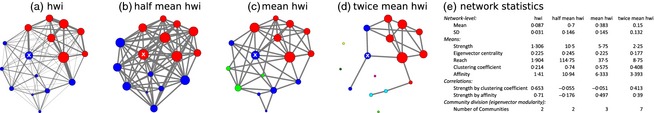
Thresholding networks can have significant impact on the structure and statistical properties of a network. (a) This network of bottlenose whales (*Hyperoodon ampullatus*), based on observations of groups of animals made at sea off Nova Scotia from 1988 to 2003 (see Gowans, Whitehead & Hooker 2001 for methodology), was calculated using the half‐weight index (hwi), to account for potentially missed observations of individuals in groups. This network was then thresholded at (b) half the mean hwi, (c) at the mean hwi (d) and at twice the mean hwi. Nodes are coloured by community (detected using leading eigenvector communities; Newman 2006) and sized by their degree (strength in the original network, binary degree in the others). This figure highlights how thresholding can lead to unpredictable results, such as individual ‘x’ changing communities, and varying relationships between node properties (such as correlations between node measures, e). For example, the correlation of individuals’ rank in terms of strength between the networks (a) and (b) is only 0·57, and the relationship has an *R* ^2^ of only 0·28.

The first consideration when setting out to collect or analyse network data is to define the relationships represented by edges in the network. Castles *et al*. (2014) highlight the importance of carefully considering the edge definition, as networks based on different edge definitions may not be equivalent. Ideally, the definition of network edges will be motivated by the biological process being investigated and edge definitions should be based upon how the animals interact and communicate. For example, studying the spread of a sexually transmitted disease requires edges that represent sexual contact. However, the technical limitations associated with collecting the data (i.e. the challenges of collecting sufficient data to generate robust estimates for a network given a specific edge definition or of studying replicate networks) must also be considered. Researchers should strive to measure the complete set of interactions (context‐specific events, such as grooming or fighting) for each dyad (two individuals that could potentially be connected by an edge). These may need to be captured using more than one network, for example one network to capture affiliative interactions and a separate network to capture agonistic interactions. However, many studies are limited to capturing information about the spatial and temporal co‐occurrences of pairs of individuals, which are called associations. If interactions are rare or difficult to observe (e.g. they occur underwater), then association networks could provide a more precise estimate of interaction rates by avoiding having many false negatives: interactions that occurred but were not or could not be observed (Farine 2015). Avoiding false negatives is important because the absence of even a few edges can significantly impact the global structure of the social network (Fig. 2 demonstrates the effects of removing weak edges). Furthermore, associations (such as co‐membership in groups) can sometimes better represent animal relationships than dyadic interactions. For example, they capture a broader range of interactions which better reflect the social environment, and therefore more appropriate when investigating broad‐scale patterns (such as measuring social selection, Farine & Sheldon 2015). Thus, when defining edges and how edge weights are calculated (Section Inferring associations from data), we recommend addressing the following questions. (i) How can the edges be made most relevant to biological process being studied? (ii) Can enough data be collected to accurately capture different relationships and the overall structure of the population (Section Estimating power and precision)?

### Edge weights and directionality

Edge values represent either the presence or absence (a binary network of 0s or 1s), or a numeric value describing the strength of the relationship or the number of interactions (a weighted network). Associations are generally symmetrical, but in many cases, interactions are not (i.e. individual A groomed individual B 10 times, but B only groomed A twice). Thus, edges can be either undirected (these are represented by at most one edge between each pair of nodes) or directed (represented by at most two edges – one in each direction). How to calculate edge weights is discussed in Section Inferring associations from data.

## Data collection methods

### Collecting interaction data

Numerous types of data can be used to construct social networks (see Chapter 3 of Whitehead 2008). A first requirement is that a substantial portion of the individuals (or each unit representing a node) in the population are uniquely identifiable. Methods for identifying individuals include using naturally occurring individual variation in coloration or morphology (Würsig & Jefferson 1990), marking individuals to make them individually identifiable (e.g. with colour‐bands McDonald 2007; Farine & Milburn 2013), or electronic tags that provide information about the location or relative distance between individuals. In addition to having individually identifiable study subjects, network analysis requires data on interactions or associations. Altmann (1974) outlined protocols for assembling raw data from direct behavioural observations, particularly focal observations, that are very relevant for animal social networks (see also Chapter 3 of Whitehead 2008).

One method frequently used to capture associations is the ‘gambit of the group’ (see Franks, Ruxton & James 2010). Gambit of the group defines all individuals within a group of animals observed at a point in time as being associated. Thus, association rates (see Section Inferring associations from data) represent the propensity for each pair of individuals to co‐occur in the same group. The fundamental assumptions of the gambit of the group are that all, or almost all, interactions of some kind take place within groups and that interactions of this type occur at a similar rate among all animals when they form groups (Whitehead & Dufault 1999; Farine *et al*. 2015, or that the group itself is meaningful to the animals. This method is particularly useful when groups of animals can be easily observed, and group membership changes over time (Silk *et al*. 2015).

### Automated techniques for collecting data

Technological advances in animal tracking are rapidly increasing the amount of data collected, both in the laboratory and in the wild (Krause *et al*. 2013). Methods include using videos to record the position of individuals (e.g. Strandburg‐Peshkin *et al*. 2013), fitting tags (e.g. Passive Integrated Transponders or PIT tags) to individuals to make them detectable when they come in proximity to logging stations fitted with antennae (e.g. Farine & Lang 2013), fitting global positioning system (GPS) devices to many individuals to capture their movements relative to one another (Godfrey *et al*. 2014; Strandburg‐Peshkin *et al*. 2015) and fitting devices that record when individuals are within a certain proximity of one another (Hamede *et al*. 2009; Rutz *et al*. 2012). With electronic tags and automated data collection, complete or nearly complete records of the associations among members of a population are sometimes available (e.g. Boogert, Farine & Spencer 2014). These technologies are typically limited to creating association (or proximity) networks, thus inferring interactions rather than capturing them explicitly. However, the amount and completeness of the data available can result in a high degree of certainty around the estimates of when individuals could have associated, despite providing little or no data on any one potential interaction.

### Attribute data

The attributes of individuals, typically consisting of phenotypic traits or details about individual state, are a vital part of social network analysis. One aim of social network analysis is to determine how sociality, or the relationship between sociality and fitness, is mediated by individual traits. Multiple traits can be captured as attributes of each node in the network, such as age, sex, size or breeding status. Some attributes may be central to the research questions, while others may be recorded because they may have an impact on an individual's network position that needs to be controlled for in subsequent analyses (see example in *Thresholding edges or individuals* in the following section).

## Inferring associations from data

### The adjacency matrix

The most fundamental data structure in animal social network analysis is the association or adjacency matrix. This is an N x N matrix, where N is the number of individuals in the study, and each cell contains the value of an edge in the network that represents associations or interactions. Typically, the matrix is read as the ‘actors’ along the rows associating or interacting with the ‘receivers’ along the columns, so that the presence/weight of the edge between individual A and individual C can be found on the first row and third column. If the network is undirected, then the matrix will be symmetrical (edge A to C is equal to edge C to A). The diagonal of the matrix contains the ‘self‐edges’, or the number of associations/interactions an individual has with itself. These are rarely used in animal networks where nodes are individuals, but can be used in networks where nodes represent other properties such as location or the agglomeration of a number of individuals, such as species (Mokross *et al*. 2014).

### Calculating edge values from observational data

Once data have been collected on the interactions or associations in the study population, pair‐wise observations can be converted into edges to populate the adjacency matrix. For interaction data, the relationship measure is often the total number of interactions observed between each pair of individuals. If the number of observations (or total time) differs between individuals, then the relationship should reflect the rates at which interactions occurred (dividing by observation time, see Farine 2015).

Association indices can be used to define edges in the network (Cairns & Schwager 1987). They typically estimate the proportion of time individuals that are associated and range between 0 and 1, where 0 indicates that they never associate, and 1 indicates that the dyad was always together. If observations are rarely missed, then the simple ratio index can be used. Here, the edge weight is calculated using: $E_{AB}=\frac{x}{x+y_{AB}+y_{A}+y_{B}},$ where the undirected edge weight between individuals *A* and *B* is the number of samples or sampling periods where they co‐occurred (*x*) divided by the number in which one or both were identified (*y*_*AB*_) is the number of times both *A* and *B* were observed in the same sample but not together, *y*_*A*_ is the number of samples where only individual *A* was seen, and *y*_*B*_ is the number of samples where only *B* was seen). If individuals are frequently missed (when they should have been observed), then the half‐weight index $\left(E_{AB}=\frac{x}{x\;+\;y_{AB}\;+\;\frac{1}{2}\;\;\left(y_{A}\;+\;y_{B}\right)}\right)$ can provide a less biased estimate of the real rate of association. Whitehead (2008) provides a comprehensive list of association indices (table 4·5, page 98) with extensive discussion and examples of their usefulness when dealing with different types of sampling bias (table 4·6, page 99). Table 2 provides an overview of some useful software packages to help generate networks, and Supporting Information Appendix S1 provides an overview of different formats to store social data.

**Table 2:** Key software packages for creating and analysing social networks

Name	Pros	Cons	Key references
ucinet/netdraw	Fully integrated point‐and‐click analyses Wide range of hypothesis testing tools Extensive documentation and help files Easily implemented network diagrams in netdraw	Requires adjacency matrix Only node randomizations Graphs are not vectorized Only available in Windows	Borgatti, Everett & Freeman (2002), Borgatti, Everett & Johnson (2013)
socprog	Fully customized for animal social networks Generates adjacency and generalized affiliation matrix from data Extensive range of tests tailored to biological questions Calculates and fits models to lagged association rates Available as stand‐alone package or as functions in matlab Free on all platforms (but see Cons) Output formats compatible with ucinet and netdraw	Detailed plotting functions available only with matlab. Compiled version only available in Windows Non‐compiled version requires matlab and Statistics Toolbox	Whitehead (2008, 2009)
r	Extremely flexible Packages provide most possible tests and network measures Animal network‐specific routines available Simple support for dynamic networks Integration of almost every possible statistical test High‐quality plotting (including GIS functionality) Free on all platforms	Steep learning curve Requires careful management of variables Requires some knowledge of software coding (see appendices of this paper)	*Animal networks* asnipe: Farine (2013a) *Network metrics*: igraph :Csardi & Nepusz (2006), sna: Butts (2008) *Dynamic networks*: time ordered: Blonder *et al*. (2012)

### Inferring edge values with automated data collection

As with observation data, edges should ideally be derived from automated data based on how the species interacts and communicates. If animals reliably interact with one another at ranges less than a particular cut‐off, this can be used to define association. However, the scale at which associations are maintained can vary over short periods of time as a consequence of different social or environmental contexts. For example, imagine a system with two pairs of birds holding neighbouring territories. When the two groups are well separated from each other (say 200 m), individuals in each group could be a relatively large distance apart from each other (say up to 20 m) and still remain in contact (e.g. acoustically). If the two groups come into territorial conflict, individuals within each group might be very close to each other (say within 1 m), but also very close to the individuals from the other group (say within 5 m). In this example, a fixed threshold of 10 m would introduce both false positives and false negatives into edge data representing spatial associations. This same issue arises when recording visits by PIT‐tagged individuals at feeders. In particular, the amount of time a flock spends at a feeder can vary greatly based on the flock size. In both temporal data‐stream and spatial proximity networks, we should not impose arbitrary thresholds if these are not clearly defined by the biology of the system (a good example is Hamede *et al*. 2009). Instead, if groups are defined as individuals being closer to each other than to others, then clustering algorithms are a useful approach.

In behavioural observations, we often implicitly define groups as sets of individuals that are closer or interact more within themselves than they do with others. Clustering algorithms, such as Gaussian mixture models (Psorakis *et al*. 2012), can extract such patterns from spatiotemporal data. These algorithms statistically infer the best‐fitting temporal boundaries of groups based on the data rather than relying on a fixed inter‐group or inter‐individual visit interval. The results are often more robust than traditional methods at capturing biological interactions (Psorakis *et al*. 2015).

### Associations and affiliations

Using association indices as edge weights produces a network that represents the pattern of association among individuals. Sometimes we are more interested in affiliations (individuals actively associating with other individuals) and wish to remove other causes of association patterns, such as spatial and temporal overlaps. Generalized affiliation indices are the residuals after regressing association indices or other measures of association (or interaction), on potential structural predictors of association, thus isolating true affiliations using a generalized linear model (Whitehead & James 2015). These generalized affiliation indices then form the adjacency matrix for a network analysis aimed at true affiliations.

### Thresholding edges or individuals

Thresholding involves either removing individuals with few observations or converting a weighted network to binary by only counting values above or below a certain value. An example of the latter is Croft *et al*. (2009), who used both high and low thresholds to investigate assortment by behavioural type. However, choosing an appropriate threshold may be problematic, and different threshold values can lead to different conclusions in the same network (see Fig. 2). Thresholding networks has been found to generate high rates of both type I and type II errors (Butts 2009; Langer, Pedroni & Jancke 2013; Farine 2014) than using the original weighted network, and should always be applied with caution. Thus, applying a threshold to an association index or interaction rate is usually not recommended other than for displaying networks (Franks, Ruxton & James 2010).

For some purposes, it is useful to remove individuals for whom there are few data. Edges connected (or not connected) to rarely observed individuals may be very inaccurate. As a result, network metrics measured for that node are unlikely to represent the true state or behaviour of the individual. This could, at worst, impact the value of every other node in the network when using global network measures. Thresholding nodes’ data should be considered on a case‐by‐case basis, but the impact of removing individuals may be smaller than the impact of having spurious edge values. A good example of thresholding individuals based on properties of the data is Aplin *et al*. (2013) who removed individuals with fewer than 100 observations as these exhibited a clear relationship between number of observations and the binary degree. In contrast, no such relationship existed for individuals with more than 100 observations.

### Creating temporal networks

There are two principal types of temporal networks, time‐ordered and time‐aggregated networks (Blonder *et al*. 2012). In a time‐ordered network, each edge is encoded with start and end times, capturing the complete set of information about when edges (i.e. interactions/associations in social analysis) occurred and their duration, and so what edges co‐occurred. Such time‐ordered data can be used to map the topological flow of information or pathogens through networks when the ordering of interactions or associations are considered to be important (Blonder & Dornhaus 2011). Blonder *et al*. (2012) provide a useful *R* package *time ordered* to perform some analyses on these types of networks.

Time‐aggregated networks, in contrast, maintain the same form as regular aggregated networks (an adjacency matrix). Here, a new network is calculated for each time slice, such as for every sampling event, week, month, season or year. Creating these networks is relatively simple as they only require the input data to be subset for each period.

Finally, a useful measure of temporal stability is the lagged association rate (Whitehead 1995). This measure calculates the probability that a given dyad is re‐observed after a given time period. This can be estimated at the network level, for different classes of individuals, or at the dyadic level. The lagged association rate is useful for describing and modelling the temporal scales over which social behaviour processes operate, or for comparing how these differ between different classes of individuals (e.g. Aplin *et al*. 2013).

## Displaying social structures

### Network diagrams

Network diagrams, formally called graphs, allow us to visualize social connections and the overall structure of the network. Nodes, usually individuals, can be represented by shapes (circles, squares) of different colours used to represent attributes, such as sex, class or gregariousness. In an undirected network, with a symmetric adjacency matrix, edges are usually drawn as a single line between each node. Relationships in a network that are directed are represented by edges with an arrow pointing in the direction of the interaction. In binary networks, edges are displayed (1), or not (0), whereas the thickness of the line is typically made proportional to the strength of a dyad's association in weighted networks. Weak edges can be omitted from the diagram to aid clarity (see Section Inferring associations from data on thresholding). In Table 3, we provide information on some of the available software packages for visualizing social networks.

**Table 3:** Other useful software

Type	Name	References
Visualizing networks	gephi	Bastian & Heymann (2009)
	graphviz	Gansner & North (2000)
	cytoscape	Shannon *et al*. (2003)
	sonia	Moody, McFarland & Blender‐deMoll (2005)
	tulip	Auber (2004)
	netdraw	Borgatti (2002)
	r package *igraph*	Csardi & Nepusz (2006)
Collecting data	j‐watcher	Blumstein & Daniel (2007)
Calculating dominance hierarchies	matman	de Vries, Netto & Hanegraaf (1993)
	codareader	Adams (2005)

### Community delineation

Some social networks are highly modular – the nodes form communities such that most edges (in a binary network) or a high proportion of the edge weight (in a weighted network) is within rather than between communities. Identifying such communities – sometimes called clusters or social units – is not trivial. Many techniques have been developed by both statisticians and network analysts, but the Newman (2006) eigenvector modularity technique is often used with animal social networks and usually works well. Many others are also available, and most of these are implemented in the R packages listed in Table 2. Also popular is hierarchical cluster analysis in which communities are embedded within one another, and the results are displayed using a tree‐like dendrogram (Whitehead & Dufault 1999). This can be an excellent representation of the social network if the society does actually consist of a hierarchically embedded set of social tiers (e.g. Wittemyer, Douglas‐Hamilton & Getz 2005), which community detection algorithms typically fail to detect. If not, dendrograms may be highly misleading (Whitehead 2008).

## Interpreting network metrics

### Types of network metrics

Network metrics are statistical measures used to characterize properties of individuals (nodes) or the whole network. Most measures are node based; they calculate a separate value for each node. Others, such as density, measure global network properties. A final type of metric are edge metrics (e.g. bridges, edge betweenness), but these are rarely applied to animal social networks and not covered further. Almost all network metrics can be expressed/defined either as a weighted measure or as a binary measure. For example, the most common nodal measures used in network analysis are binary degree and strength, which are the sum of the number of edges each node has or of all the edge weights connected to it, respectively. The average or distribution of the measures can be used to describe more general properties of the social structure. For example, one of the fundamental properties of networks is the degree distribution (see pp. 243–260 in Newman 2010).

We give definitions of some of the most commonly used network metrics in Box 2, Newman (2010) provides detailed accounts of many more, and Borgatti (2005) provides detailed a detailed account of their appropriate use. Network metrics can easily be calculated in most network analysis programs, including *socprog* (Whitehead 2009) and *ucinet* (Borgatti, Everett & Freeman 2002). Further, there are some excellent libraries in *r* (R Development Core Team 2013), in particular *igraph* (Csardi & Nepusz 2006) and *sna* (Butts 2008) which calculate almost all common network algorithms (see Table 2).

Box 2 **Common network metrics**

B = binary only, W = weighted only, BW = weighted and binary networks.

**Node‐level metrics**

Degree (B): the binary degree is the count of the number of edges connected to the node. If a network is directed, degree can be the partitioned into in‐degree and out‐degree, representing the number of incoming and outgoing edges, respectively. This measure captures the gregariousness of individuals, in terms of the number of associates or interaction partners.

Strength (W): the weighted equivalent of binary degree is the sum of all edge weights connected to the node (if all edges have a weight of 1, then the strength will equal the binary degree). Strength can also be partitioned into in‐strength and out‐strength for directed networks. This measure typically represents the expected total interaction or association rate per sample. For example, a node with a strength of 2 would be expected to be observed with approximately two other individuals on average (if using most association indices).

Betweenness Centrality (BW): a count of the number of shortest paths that flow through the node. This measures how important a node is for connecting disparate parts of a social network. Individuals with a high betweenness centrality are likely to connect largely independent communities. This often highlights individuals that have a greater tendency to change groups than others.

Eigenvector Centrality (BW): the sum of the centralities of an individual's neighbours. High centrality can be achieved by having either a large degree or being connected to associates with a high degree (or both). This is a useful property as it captures the potential ‘importance’ of individuals in the network, as social hubs, or for the propagation of information or diseases through animal populations.

Page Rank (BW): a robust measure of centrality for directed networks that divides the centrality gained through associates by the associate's out‐degree. This means that very central nodes only pass on a small amount of centrality to each node that is connected to them, thereby controlling the measure of eigenvector centrality for long tails in the degree distribution. Individuals with a large page rank are disproportionately important for connecting different components of the network, and this measure is likely to be important when investigating flows through networks.

Reach (BW): Measure of what proportion of all other nodes can be reached in one step, two steps and so on. This is the equivalent of calculating how much of the network is in ‘n degrees of separation’. This measure has not been explored much in animal social networks, but could be useful for investigating differences in social structure, or implications of changes in social structure, when conducting experiments (such as removals of key individuals). It is also likely to be interesting in models of disease or information transmission in order to estimate how quickly most individuals in a population can become infected/knowledgeable.

**Network‐level metrics**

Density (BW): the number of edges in a network divided by the total possible edges (B), or the sum of edge weights divided by the number of possible edges (W). A potentially important measure for normalizing observed degree distributions as larger networks tend towards very low densities.

Homophily/Assortativity (BW): the correlation in the phenotype of connected individuals. Positive assortment suggests that nodes are more connected than expected, whereas negative assortment suggests avoidance of alike nodes. This can now be measured on weighted networks and is a powerful approach for identifying phenotypic structure in social networks. For example, positive assortment by degree (gregariousness) has been linked with rapid spread of information or disease through social networks.

Transitivity (BW): the proportion of triads (trios of nodes) that have three edges divided by the number of triads that have two edges. When compared to null models, this identifies whether trios have a tendency to be more or less connected than expected. This is potentially an important measure, particularly when measuring interactions, as it captures the level of clustering in the network. For example, grooming networks may have low transitivity if grooming is directed up or down a linear hierarchy. Transitivity can be measured for nodes as well as at a network level. However, care should be taken if using the gambit of the group approach to capture associations, as this automatically closes triads (but the impact of this remains unexplored).

### Interpretation of network metrics

Studies use network metrics to estimate differences between individuals in their placement within a social network. In animal networks, the most common are based on measures of centrality. This generally refers to individuals that are broadly more (or more strongly) connected than others. We recommend caution when interpreting network metrics, as these depend on both the measure used (e.g. degree vs. betweenness) and the edge definitions (e.g. rates vs. number of interactions), and on how the population is structured (e.g. if a population is structured into communities, metrics calculated within a single community may be very different from those calculated for the entire population). For example, the strength (weighted degree) of individuals in a network with edges defined as association indices defined using group membership is roughly proportional to their average typical group size (Jarman 1982). In contrast, the strength of a node in a network using counts as edge values simply represents the total number of interactions observed from that individual. Box 3 provides an example of how the network structure can impact the interpretation of sets of centrality metrics. Because the metric can have different interpretations on different social networks, we recommend visualizing the structure of the network and the correlation structures between different metrics. We also discuss why comparing metrics across different networks is also problematic in Section Remaining challenges and future directions.

Box 3 **The effect of network structure on network metric correlations**

The structure of the network can impact the correlation between different measures of centrality. While in many cases, centrality measures might capture the same biological processes, in some other cases, these might differ. In Box 3 (Figure), we present two toy networks. The first (a) contains two clusters joined by a single individual. In this network, node 1 provides a bridge between the two clusters, and it has a high betweenness (b). However, node 1 has the lowest degree. Thus, if we were to investigate dynamics of spread, betweenness might provide a better estimate of relative node importance. The second network (c) contains individuals that are more uniformly connected. Individual 1 in this network has both the highest degree and, by far, the highest betweenness. VanderWaal *et al*. (2014) developed a metric called cut point potential (the potential for individuals to disconnect parts of the network if they are removed) to disentangle the effects of degree from betweenness, and this may warrant more widespread use. In both networks, eigenvector centrality and page rank are both strongly correlated with degree (a rank correlation close to 1), whereas clustering coefficient is not (and rarely is). Thus, when interpreting the relative importance of nodes in a network, the relationships between different network metrics may be informative. The node‐level ratio of network metrics could also be useful (such as the ratio of degree to betweenness), though to our knowledge, this has not been explored.

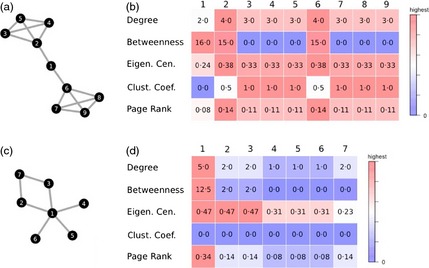

## Constructing null models

### What are null models?

Null models are data sets that are based on observed data, but generated in a way that allows some aspects of the data to be random. This can involve generated new data using simulations inspired by the observed data (e.g. creating random networks with the same degree distributions), but more commonly involve shuffling existing data to create expectations of random given certain constraints. Many potentially realistic null models can be tested using social network analysis. For example, one null model might assume that individuals have no preferred affiliates given their spatial and temporal use of the study area, or another that there is no tendency of males to preferentially associate with other males given the overall gregariousness profiles of members of each sex. Note the ‘given …’ clauses – these are what make hypothesis testing on social networks so challenging. Hypothesis testing on networks generally relies on null models constructed by randomizing the data. The key issue is whether the null model actually represents the biological null hypothesis being tested.

### Using null models accounts for non‐independence

A key consideration when applying a statistical test to a social network is non‐independence in the data. Network measures are inherently non‐independent and thus violate the assumptions underlying most parametric statistical tests (Croft *et al*. 2011b). In particular, each association index is shared by two individuals. This results in over‐inflation of the degrees of freedom used to calculate significance (see Appendix S2 for an example). Randomizations are the most widely used approach to control for non‐independence (Croft, James & Krause 2008; Whitehead 2008; Croft *et al*. 2011b). One strength of randomizations is that they can be integrated with almost any statistical test.

Another source of non‐independence is temporal structure. For many analyses, the assumption is that different samples of data (i.e. observations) are independent. Because of the persistence of associations and the autocorrelation of interactions, this is often not the case. For example, data collected by following a focal animal for a defined period of time will be pseudo‐replicated. Using sampling periods, where data is aggregated to generate independent samples, is one way of reducing the effects of temporal partitioning (e.g. by placing all data from one focal follow into one sampling period). Lagged association rate analyses (Whitehead 1995) can help determine a suitable sampling period for which subsequent samples can be considered independent. Null models are useful for dealing with this issue by randomizing blocks of pseudo‐replicated data, thus generating a realistic null distribution when estimating significance with statistical tests such as regressions (see next section).

### Randomization techniques

Data randomizations use the observed data to generate replicated data sets, where each replicate is a shuffled version of the original data (Manly 1997). In each step in the randomization, a new network is created with the same nodes as the original, but with randomized edges based on assumptions about relationship under investigation. Repeating this process many times creates a distribution of values that represents the expected relationship under the assumption of the null hypothesis. The process of defining a null model (how the data will be shuffled) involves keeping certain aspects of the data the same during each randomization step. This has two functions: it controls for particular factors that could influence the data (such as the number of observations of each individual or the spatial distribution of individuals), and it provides a framework that facilitates the comparison of different hypotheses (Gotelli & Graves 1996). A common null model is to randomize who associates with whom, but to restrict swaps between pairs of individuals observed in the same location at the same time. Significance is then estimated by comparing the observed statistic to the distribution of same test statistics measured on the randomized networks. We provide an overview of how to integrate randomizations into hierarchical models in Box 4, and worked examples in Appendix S2.

Box 4 **Incorporating null models into hierarchical models**

The application of randomizations is not limited to traditional permutation tests. They can also be used to control for confounding factors in more traditional tests and calculate appropriate *P* values. To demonstrate how to calculate *P* values in a GLMM using randomizations, we generated a simulated network consisting of 40 individuals from two different sampling areas (the code is contained in Appendix S3). In this simulation, we started by generating a Poisson‐distributed gregariousness score for each individual, with individuals in area 2 having slightly higher average gregariousness. We then allocated a sex to each individual, with the probability that an individual is male that is proportional to the individual's gregariousness value. Using the *rgraph* function in the *R* package *sna*, we then simulated 100 sampling periods. In each sampling period, the probability that two individuals interacted (had an edge) was proportional to their combined gregariousness scores (but individuals in different areas did not interact). We then generated the networks (Figure Box 4a; males red, females blue, node size is proportional to strength) using the simple ratio index, finding that the degree was higher in males than in females (Figure Box 4b). Fitting the GLMM model, *Strength ∼ Sex + (1¦Area)* using the *lmer* function in the *lme4* package generated a positive coefficient value (coefficient ± SE = 2·598 ± 0·787, *t* = 3·302, see Box 4 Table 1). To calculate the *P* value, we then performed 1000 data‐stream permutations (swapping observations in the sampling periods), controlling for time (sample) and space (area). After each swap, we re‐fit the same model but with the strength value of each node calculated from the newly created (and increasingly random) network: *Strength*_*i*_ *∼ Sex + (1¦Area)*, where *i* is the current randomization. Comparing the observed coefficient value (2·598, Figure Box 4c red line) with the distribution of the 1000 coefficient values from the permutations (Figure Box 4c, black histogram) captured this significance of this difference (*P*_rand_ = 0·035).

**Table:** 

Fixed effects	Coefficient	Standard error	*t* statistic
*Intercept*	4·597	0·909	5·062
Sex (male)	2·598	0·787	3·302

Random effect	Variance	Standard error	
Area	0·901	0·953	
Residual	5·895	2·428

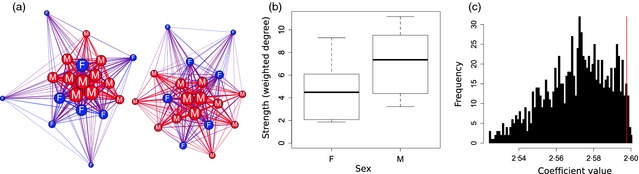

There are two main approaches to building null models for animal networks: node‐based randomizations and data stream‐based randomizations. Node‐based randomizations involve entirely re‐distributing the attributes of the nodes in the network, while maintaining the same number of each type, when creating each random network (Croft, James & Krause 2008; Whitehead 2008; Croft *et al*. 2011b). This randomization is often used to test for differences in network position between nodes with different attributes (do males have more associates?). It is simple to implement as it relies only on the adjacency matrix. However, this cannot control for parameters other than the number of individuals of each type. Further, it relies on the assumption that the observed network is a strong representation of the true network (Croft *et al*. 2011b), and has the potential for much higher rates of type I and type II errors than randomizations based on shuffling the data stream (Farine 2014).

Permutations of the data stream involve sequentially swapping observations between individuals (Bejder, Fletcher & Brager 1998). Data‐stream permutations can be used to test the same hypotheses as node‐based randomizations. They can also be used to test for preferred and avoided relationships, either overall or between specific dyads. Swaps can occur at the group level (A is moved from group 10 to group 15, and B is moved from 15 to 10), or at the dyadic level (groomer A is swapped from grooming C to grooming D, B is swapped from D to C). This method is very powerful as it can control for a number of different possible confounding effects, such as controlling for spatial or temporal variation in the presence of individuals (Whitehead 1999; Whitehead, Bejder & Ottensmeyer 2005; Sundaresan, Fischhoff & Dushoff 2009), and for the sampling method by keeping the number of observations of each individual fixed. We recommend always using data‐stream permutations when possible.

## Hypothesis testing in animal social networks

### Determining if the network is ‘non‐random’

Often, we want to test whether a network is more structured than expected from random. To do so, a test statistic needs to be chosen that will be used to compare a property of the observed data to the same property measured on a set of randomized networks. This statistic should represent an aspect of the structure that is expected to differ between non‐random and random networks. For example, if individuals have preferred and avoided relationships, then the standard deviation of their association strengths should be higher in the observed network than in a network where individuals associate with others at random (and thus equally). Whitehead, Bejder & Ottensmeyer (2005) propose different test statistics for different null hypotheses, such as the lack of short (within sampling periods) and long (between sampling periods) preferred relationships among dyads, or uniform gregariousness among individuals. The most easily interpretable test statistic is the coefficient of variation (CV) of the association indices. This measure has the added benefit of solving the problem of presenting effect size, as its values on the observed network and the mean of the randomized ones indicate how strong the effect is. To test if the network contains more preferred/avoided relationships than expected at random, the CV of the observed network is compared to the CV measured in 1000 or more randomized version of the network (see previous section). The *P* value is then calculated by taking the number of times the CV value of the observed network is smaller than a randomized network, divided by the number of randomizations (see Ramos‐Fernandez *et al*. (2009) and Mourier, Vercelloni & Planes (2012) for good examples of this method being applied).

### Using network data in linear and hierarchical models

Linear models (in particular generalized linear models, GLMs, and generalized linear mixed models, GLMMs) are an attractive framework for investigating relationships between an individual's attributes (phenotype) and its network position (social network metric). This approach can easily control for confounding effects arising from data sampling (e.g. including the number of observations as a fixed effect or using a binomial model that includes both the numerator and denominator of the association index). While GLMMs deal well with repeated observations made on individuals, they cannot readily control for non‐independence in the network measures themselves (Snijders 2011). In Box 4, we provide an example of combining GLMM with network randomizations. In Box 5, we show how this approach deals with biases that can easily arise when collecting social data (see *Is there a bias in the data sampling?* in Section Estimating power and precision). When using this method, a table with the coefficient values, standard error, test statistic (e.g. *t*‐value) and the *P* value calculated from the randomization test can all be reported. The study by Boogert, Farine & Spencer (2014) is a good example of this framework applied to testing hypotheses on social networks.

Box 5 **Demonstrating how permutations deal with biases**

A major potential source of error in social networks is sampling bias. If there are differences in how observable individuals are as a function of their class, this can easily lead to spurious results. We demonstrate this by repeating the simulation from Box 4, but this time males and females do not vary in gregariousness (we maintain the same distribution, but now allocate sex randomly). After generating sampling periods, we now allocate a probability that each female is observed with only 70% reliability, whereas bright conspicuous males are observed with 100% reliability (the code for this simulation is found in Appendix S4). Thus, even though both classes had the same average degree measured on the full set of associations (Figure Box 5a), reducing the observation probability of females introduced a difference in their means (Figure Box 5b). Fitting the same GLMM model as in Box 4 suggested that this difference was highly significant (Table Box 5, note the *t*‐value is larger than in the Box 4 example). However, when calculating the *P* value using randomizations (see details in Box 4), the observed coefficient value (2·143, Figure Box 5c red line) did not fall outside the distribution of randomized coefficient values (Figure Box 5c histogram), resulting in a non‐significant effect (*P*_rand_ = 0·226). This example highlights how using randomizations improves our ability to capture biological patterns in the *real* rather than in the *observed* network.

**Table:** 

Fixed effects	Coefficient	Standard error	*t* statistic
*Intercept*	4·315	0·621	6·949
Sex (male)	2·143	0·578	3·707

Random effect	Variance	Standard error	
Area	0·371	0·609	
Residual	3·186	1·785

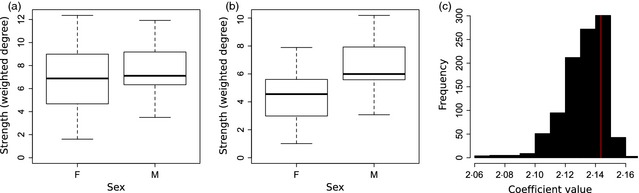

### Mantel tests and MRQAP

Sometimes we wish to test the hypothesis that association strength, or interaction rate, is related/unrelated to some other dyadic measure, such as genetic relatedness or gender similarity. The Mantel test (Mantel 1967) makes such a test, usually by means of node‐based permutations. There are a number of nonparametric versions of the Mantel test which may be appropriate especially with interaction numbers or rates, which can have high skew (e.g. Hemelrijk 1990). The matrix correlation coefficient (i.e. the correlation between the corresponding non‐diagonal elements of the two matrices) is a suitable effect size measure for Mantel tests.

Building on the Mantel test, we can test for a relationship between a dependent dyadic variable, such as association strength, and an independent variable, such as genetic relatedness, while controlling for one or more further independent variables, such as gender similarity or home range overlap. In this case, regressions can be performed on the matrix data using a procedure called multiple regression quadratic assignment procedure (MRQAP). There are several ways that the MRQAP permutations can be performed, but both theory and practice favour a recently developed double‐semi‐partialling technique, which randomizes the residuals of a regression model rather than the independent or dependent matrices themselves (Dekker, Krackhardt & Snijders 2007). Partial correlation coefficients are suitable effect size statistics for MRQAP tests. The study by VanderWaal *et al*. (2013b) is a good example of this method being used to test the structural similarities between association and pathogen transmission networks.

Unfortunately, all implementations to date rely on node‐based permutations of the data, and the validity of using data‐stream permutations with MRQAP has not, to our knowledge, been investigated. An alternative, proposed by Rushmore *et al*. (2013), is to fit edge weights into a logistic mixed‐effect model, and this could then be combined with an appropriate null model (see previous section).

### Network‐based diffusion analysis

An increasing number of studies are investigating the role of social networks in mediating population‐level processes, such as the spread of information. Network‐based diffusion analysis (NBDA) is a powerful tool for inferring the relative rates of social transmission of information in a social network and rates of individual‐based learning (Franz & Nunn 2009). NBDA uses the order or times when individuals were observed to have acquired some information to fit parameters of social transmission and non‐social learning based on their network links to individuals that already have the information (Hoppitt, Boogert & Laland 2010). Hoppitt & Laland (2011) provide a useful manual and *R* package for using NBDA. They include details on how to incorporate confounding effects into the analysis. Farine *et al*. (2015a) and Nightingale *et al*. (2015) also provide important extensions to the NBDA framework. These deal with the need to compare competing networks that facilitate diffusion (i.e. different hypothesized relationships), and with estimating the uncertainty surrounding the estimation of the independent network. Because NBDA has an inherent null hypothesis (no social transmission), it does not need to be combined with randomizations, although the potential for integrating alternative null models (such as data‐stream permutations) into this method has yet to be explored. The *s*, or rate of social transmission, parameter can be used as an effect size and reported with 95% confidence intervals. NBDA has been widely used to investigate transmission of behaviours in animals, for example in fish (Atton *et al*. 2012), cetaceans (Allen *et al*. 2013), primates (Kendal *et al*. 2010; Hobaiter *et al*. 2014) and birds (Boogert *et al*. 2008; Aplin *et al*. 2012, 2015; Farine *et al*. 2015).

### Multiple hypothesis testing using null models

In addition to using randomizations for null hypothesis testing, multiple null models can also be used to evaluate competing hypotheses (Gotelli & Graves 1996). This involves keeping different aspects of the data constant in each model to identify whether they affect social structure (Farine 2013b). For example, to test whether an observed phenotypic structure in a social network is driven by spatial distribution of individuals or by social avoidance, we can build one null model that controls for space and one that does not. If both null models are rejected, this suggests that the observed patterns are driven by social attraction/avoidance because both null models create more random networks than observed. In contrast, if the spatially controlled null model is not rejected, but the non‐spatial model is, then individuals are non‐randomly distributed in space, but we cannot reject the hypothesis that they associate randomly with respect to phenotype within their location. This approach is demonstrated in Farine *et al*. (2015b).

### Simulations

Simulations can be used to infer the mechanisms that underpin network formation or to explore the consequences of network structure for population processes. There are two types of simulations that are useful in network studies: data‐driven simulations and fully simulated networks. Data‐driven simulations involve building an agent‐based model (Railsback & Grimm 2012) in which patterns of interactions are determined by the observed network. This can be used to investigate how social structure mediates population processes, such as the spread of a disease in that population. Alternatively, individuals (nodes in the network) can be removed to determine the impact on social connectivity and network robustness (e.g. Manno 2008; Wey *et al*. 2008). To our knowledge, no study has performed simulations on increasingly random networks (using data‐stream permutation methods) to investigate whether some aspects of population processes are conserved under different null models, or how fast these processes are expected to degrade.

The second approach stochastically simulates behaviours of individual actors based on a minimal set of rules, or mechanisms. Comparing the result of simulations to empirical data is a powerful way of inferring candidate mechanisms generating social structure (Cross *et al*. 2012; Sumpter, Mann & Perna 2012; Farine, Downing & Downing 2014). For example, simulations could be used to test whether females preferentially copulated with successful males by comparing the degree distribution from an observed network with the degree distributions from simulated networks that implement preferential mating.

### Reporting statistical results

A criticism of null hypothesis significance testing is the presentation of ‘naked *P* values’ (Anderson, Burnham & Thompson 2000) without any indication of the size of the effect being tested. The results of hypotheses tested on social networks are often presented without effect statistics, but these should be reported. In permutation tests, if the test statistic is directly interpretable (e.g. the standard deviation of the association indices or the coefficient values of a model), the effect size can be reported by giving the value measured on the observed network as well as the mean or 95% range of the values measured on the permuted networks. This provides information both on the size of the effect and on the uncertainty associated with the conclusions drawn from the result.

## Estimating power and precision

### Have enough data been collected?

Clearly, the more the data available, the more the observed network will mirror the real social network of the animals, and the more powerful will be tests against null hypotheses. Whitehead (2008) provides a guideline for estimating the sampling effort required to achieve a reliable social network (defined as a correlation between the edges of the real and the observed network of at least 0·8). A network that is moderately socially differentiated, where the coefficient of variation (CV) of edge weights of the real network is approximately 0·2, requires a mean of about 50 identifications per dyad. This decreases as the network becomes more strongly differentiated, for example as relationships become less mixed and start to resemble pairs forming territories. A highly differentiated population (with a CV of approximately 0·6) requires an average of five identifications per dyad, whereas an extreme population (CV of around 10) requires only 0·02 identifications per dyad (i.e. there is high certainty that a single observation of an edge is accurate). The power of permutation tests to reject null hypotheses is highly dependent on the strength of the pattern being tested and the amount of data collected (Whitehead 2008). In most cases, large data sets are required, a further reason why automated data collection systems have become very useful.

Realistically, studies are often limited either by resources or by logistics. Given this, it can be useful to identify strategies that will maximize the quality of the data that are collected (see Estimating the quality of an observed network). When sampling effort is limited, the simulation study by Franks, Ruxton & James (2010) suggests that increasing the number of censuses, rather than increasing the proportion of individuals sampled in each census, generates a more robust network when sampling fission–fusion groups. This may also be relevant when sampling populations with stable social groups, in which case collecting more samples from fewer groups may yield better results than sampling more groups.

### Dealing with missing individuals

The issue of missing individuals need to be assessed in terms of the biological question under investigation. The simulation study by Silk *et al*. (2015) suggests that the correlation between node‐level metrics measured on partial and full networks has a relatively linear relationship with the proportion identified. However, sampling only a small proportion of the population may have a greater negative impact on network‐level measures, such as phenotypic assortment (see Farine 2014), than on means of pair‐wise measures. The issue of how to deal with missing individuals in a social network is an outstanding question. To our knowledge, there is no way, other than using simulations, to estimate how well a network based on a subset of the population captures the properties of the real network.

### Is there a bias in the data sampling?

Perhaps a more important issue than missing individuals is biased sampling. There are many ways that sampling may bias observations towards some individuals which become disproportionately represented in the data. For example, brightly coloured or more active individuals may be easier to find, and these individuals may be observed more often. In many cases, bias is inevitable, and these biases need to be considered either in quantitative analyses or when discussing the results. In the case of the bright individuals, we may expect that if these are observed more often than others, then if there was no difference in their real gregariousness, these individuals could still have more associates (edges) in the social network and therefore greater network centrality. Currently, the best ways to deal with these situations are to use generalized affiliation indices (Whitehead & James 2015) or to build null models using permutations that account for these sampling differences (see Box 5).

### Estimating the quality of an observed network

How to estimate whether an observed network is robust and precise remains an outstanding question. Above, we have provided some guidelines to estimate how well a population should be sampled, and what to prioritize during data collection. If data have already been collected, Lusseau, Whitehead & Gero (2008) suggest using bootstrapping (resampling data at random with replacement) or jackkniffing (removing a certain percentage of data) as a way to estimate the confidence intervals around network measures. Wey *et al*. (2008) adapted jackkniffing, combined with anovas, to calculate the percentage of data that could be removed without causing a significant change in the different network measures. In their case, they found that network measures were robust up to 75% of data removed. Finally, Cross *et al*. (2012) provide a useful framework using hierarchical models to evaluate the relative importance of difference factors (individual, dyadic and environmental) driving variation in association rates in an observed network. However, more research is required (using mathematical models, simulated networks or very well‐sampled networks) to determine the following: (i) whether such approaches provide a good estimate of the potential error in an incompletely sampled network; (ii) how confident we are that the network we observed is a good estimation of the real patterns.

### Controlling for multiple comparisons

One criticism of many social network studies is that they often examine the relationship of multiple metrics with multiple individual‐level attributes (e.g. traits or pathogen status). With many significance tests, type I errors multiply, an issue that taxes statistical analyses generally. Bonferroni and other corrections can be used to reduce the type I error rate, but at the expense of type II errors. In network analysis, we recommend concentrating on effect sizes rather than *P* values whenever possible, thus avoiding the multiple comparison problem.

## Remaining challenges and future directions

### Comparing networks and comparative studies

Comparing networks across contexts (e.g. between populations or species) remains one of the main challenges in network analysis (see Chapter 7 in Croft, James & Krause 2008). The lack of measures that can be used to make robust comparisons among networks prevents their use in comparative studies to investigate broad questions in social behaviour. Comparing networks is challenging both because measures are influenced by network topology (e.g. degree distributions by the number of nodes in a network; how discrete or modular is the population being studied) and the lack of standardization in data collection. If data collection is completely standardized, for example all individuals in two populations of similar size are sampled at a high‐resolution and in the same context, then network comparison may be possible. This is because differences arising from the network structures are attributable to biological processes. Thus, we suggest that issues with comparing networks should not frighten biologists from collecting data in properly replicated networks. In contrast, if data are collected differently, such as by using focal follows vs. gambit of the group, then the methods themselves may generate fundamental differences in network structure that are not related to the biology of the organisms under investigation.

### Dynamic network analysis

Temporal dynamics represents a significant analytical challenge in social network analysis. First, the data must be collected and analysed at a scale that is appropriate for the biological questions (Pinter‐Wollman *et al*. 2013). This includes collecting sufficient data to generate a representative network at each point in time. Another major obstacle is the development of robust statistical tests for dynamic networks, including appropriate null hypotheses and associated randomization‐based null models. For example, to test whether the property of a network (such as mean degree) is increasing more than expected over time requires quantitative null hypotheses that are based on previous knowledge of the system. Hobson, Avery & Wright (2013) provide a useful framework highlighting different scales at which temporal information can be incorporated into network analysis. Computer scientists are also rapidly developing tools for analysing temporal networks, such as dynamic community analysis (Tantipathananandh & Berger‐Wolf 2011), though these may take some time to filter down to biological users and in their current formulations, they are not realistic for many animal social networks.

### Repeatability of network position

Whether individuals have consistent network positions across different samples (i.e. repeatability) may be a critical consideration when exploring social factors that mediate individual fitness (Wilson *et al*. 2013). An increasing number of studies are demonstrating that network position is repeatable across different samples (Boogert, Farine & Spencer 2014; Jacoby *et al*. 2014). Attempts to understand the determinants – ecological, behavioural or genetic – of individual network properties should begin with an exploration of the stability of these properties across repeated observations of individuals. Nakagawa & Schielzeth (2010) describe a framework for measuring repeatability based on repeated measurements made on the same individuals, which with repeatedly sampled networks can determine whether individual network positions are repeatable. This framework uses GLMMs to calculate the proportion of variance in the distribution of network measures that is attributable to the individual identities (as a random effect) in the network. Repeatability may also be a useful measure of robustness. For example, data could be partitioned into two samples and the rank correlations of individual network metrics measured. Both these methods estimate the consistency of individual positions and network structure. This may be an important assumption to examine when testing hypotheses based on relationships such as the link between network centrality and reproductive success.

### Using networks in an experimental framework

Most published social network studies still remain largely descriptive. This may potentially result in over‐interpretation of the strength attributed to published findings in a given field (James, Croft & Krause 2009). For example, different studies may observe similar patterns arising from different mechanisms (or worse, from having similar biases in their observation data). Thus, there is a pressing need for studies that combine network analyses with experimental manipulation of animal groups that explicitly confirm the results of observational studies and identify underlying mechanisms. This can be done by experimentally removing individuals, where the network is used to identify key mediators of group structure (such as in Flack *et al*. 2006), or by altering ecological conditions experienced by groups. The latter has generally been restricted to natural experiments, such as measuring community structure in groups across habitat remnants of different size (e.g. Mokross *et al*. 2014). An exception is Firth & Sheldon (2015) who experimentally controlled individual's access to different food resources to quantify the impact of spatially breaking‐up flocks of birds. They found that new network connections formed under this regime were carried over into other contexts. Another experiment introduced novel behaviours into replicated animal populations to track the spread of information through networks (Aplin *et al*. 2015). Finally, Croft *et al*. (2011a) experimentally manipulated parasite loads and found that infected fish were actively avoided by conspecifics. However, controlled manipulation experiments remain rare. Yet, they are critical for understanding the role of individuals and social structure in social dynamics, or what causes individuals to have different network positions. Only through experiments will social networks be able to provide definitive causative evidence for socially mediated mechanisms underpinning evolutionary processes.

## Conclusion

Studies of animal social networks can break new ground across a broad range of disciplines. This may require an increased reliance on experimental manipulations, repeated sampling of individuals across individuals’ lifetimes and network analyses that move beyond dyadic measures. Combining observed data with simulation models is a promising avenue to quantitatively assess competing mechanisms. For example, this approach could be used to discover how the rules that govern processes such as group joining and leaving drive social structure. In turn, this may, in the future, help to inform management or conservation action. In all cases, network approaches will be improved by defining biologically appropriate network edges, ensuring well‐sampled networks, including robust null models in statistical testing, and evaluating the uncertainty surrounding the results.

## Data accessibility

The code for the simulations in Boxes 1 and 2 is contained in Appendices S3 and S4. The bottlenose whale network (Fig. 2) is available as Data S1.

## Supporting information


**Appendix S1.** Data formats.
**Appendix S2.** Performing basic network analyses.
**Appendix S3.** Simulation code for Box 4.
**Appendix S4.** Simulation code for Box 5.

**Data S1.** Association matrix of bottlenose whales (*Hyperoodon ampullatus*) observed at sea off Nova Scotia from 1988 to 2003.
