# Supplementary material for: Constructing, conducting and interpreting animal social network analysis
Source: J Anim Ecol. 2015 Aug 11;84(5):1144–63. doi: 10.1111/1365-2656.12418 (PMC4973823; doi:10.1111/1365-2656.12418)
Supplement: Supplementary file 1 — Appendix S1. Data formats. Appendix S2. Performing basic network analyses. Appendix S3. Simulation code for Box 4. Appendix S4. Simulation code for Box 5. [file JANE-84-1144-s001.pdf]

Appendices for: Constructing, conducting, and understanding social network analysis

Damien R. Farine and Hal Whitehead

Edward Grey Institute, Department of Zoology, University of Oxford, South Parks Road, Oxford OX1 3PS, UK

Smithsonian Tropical Research Institute, Ancon, Panama

Department of Anthropology (Evolutionary), University of California Davis, 1 Shields Ave. Davis, CA 95616, USA

Department of Biology, Dalhousie University, 1355 Oxford St, Halifax, Nova Scotia, Canada B3H 4J1, Canada

Appendix 1: Data formats

1.1 Working with attribute data in R

Managing data is one of the most important skills to develop when learning to use R. Unlike using a spreadsheet program, where the data is always visible, R can encapsulate data into variable names, through which data can be accessed directly. Working with network data typically requires the use of several different types of data formats, including data frames, matrices, and scalars. In this section, we provide an example in which we will construct a toy network by manually entering data into a new R session.

A *scalar* is simply a variable that contains only one number. When that variable name is used, it is then automatically replaced by the value it contains. For example, it is good practice to store the total number of individuals in a study as a variable. Using this variable, instead of typing the number each time it needs to be used, makes the code more re-usable since any changes in the population size (or if using the code for a different study) means that the number only has to be updated once. Below we open a new R session, and define a variable for a population size of 12. We then use this variable to calculate the number of potential edges in an undirected network.

```
> N <- 12
> N*(N-1)/2
```

```
[1] 66
```

Variables can also store more than one number. These are typically referred to as *matrices* or *arrays*, and can be n-dimensional. A one-dimensional matrix might typically contain information that relates a single variable to each node in a network, such as the age of each individual (each node in the network). The simplest way of defining these with existing data is to use the *c* function. Empty one-dimensional matrices can be defined using the *rep* function. Note that the help file for any function in R can be accessed by preceding its name with a question mark: *?rep*. Below we create a variable to contain all the age of each individual in a network, and a variable of the same size (i.e. with the same number of cells) that we fill with values from 1 to 12 (providing a unique number for each individual).

```
> age <- c(1,1,1,2,3,4,4,4,4,5,5,5)
> id <- seq(1,12,1)
```

A unique feature of R is the built-in ability to have different types of data in one variable, called a data frame. This is incredibly useful when storing all of the variables associated with each node in a network, for example size, sex, age, etc. Here we combine the above information about each individual into a data frame with a new column containing the sex of each individual. In this same function, we could also add each individual's ID, but add it in the next line to demonstrate how columns can be created dynamically in data frames.

```
> sex <- c("Male","Male","Female","Female","Female","Male","Male",
        + "Male","Female","Female","Female","Male")
> attribute_data <- data.frame(ID=id, Age=age, Sex=sex)
> attribute_data$ID <- id
> attribute_data
   ID Age    Sex
1   1   1   Male
2   2   1   Male
3   3   1 Female
4   4   2 Female
5   5   3 Female
6   6   4   Male
```

```
7   7   4   Male
8   8   4   Male
9   9   4 Female
10 10   5 Female
11 11   5 Female
12 12   5   Male
```

Data capturing the associations between individuals in a network are best stored in a multidimensional variable (usually a matrix). Before defining a network to accompany the network attributes we created above, we first provide some background on how to use matrices and n-dimensional arrays in Appendix 1.2, and return to this example in Appendix 1.3.

1.2 Matrices and multi-dimensional arrays in R

Most data associated with networks will be either two or three dimensional. A two-dimensional matrix is the equivalent of a single spreadsheet, while a three-dimensional matrix is the equivalent of multiple tabs in a spreadsheet (but where the data in each tab has the same number of rows and columns). This is very useful as it enables data to be accessed using only a single variable. For example, if a dynamic network had data for one week at an hourly resolution, this could potentially require up to 168 tabs in a spreadsheet (which would be un-manageable and too easy to make mistakes). In contrast, all of this information can be stored in a single variable in R. The data in these matrices can be accessed in a number of ways, and the simplest is using square brackets ([and]). Importantly, when the indexing into a matrix, the part within the square brackets must include information for all of the dimensions of the variable (separated by commas). Leaving a space between commas for a dimension will return every possible value in that dimension (for example every row). Below are a few commented examples of two and three-dimensional matrices (the hash symbol precedes comments) and how to access different parts of the data within them:

```
# Define an N x N matrix filled with zeros
> my_2Dmatrix <- matrix(0, nrow=N, ncol=N)

# Assign the value 10 to the cell in row 5 and column 9
> my_2Dmatrix[5,9] <- 10

# Look at the values of all cells in row 5
```

```
> my_2Dmatrix[5,]
 [1]  0  0  0  0  0  0  0  0 10  0  0  0

# Define a 2 x 2 x 3 matrix, using array, that contains three 2x2 2-D matrices
# filled with zeros, Note that here we provide a 1D matrix to the 'dim'
# argument in this function that contains the size of each dimension (2 by 2 by 3)
> my_3Dmatrix <- array(0, dim=c(2,2,3))

# Assign the value 5 to each cell in the column of the third matrix
# Note that leaving the row value blank accesses all rows in that column.
> my_3Dmatrix[,2,3] <- 5

# Look at the entire data (avoid doing this if you have large matrices!)
> my_3Dmatrix
, , 1

     [,1] [,2]
[1,]    0    0
[2,]    0    0

, , 2

     [,1] [,2]
[1,]    0    0
[2,]    0    0

, , 3

     [,1] [,2]
[1,]    0    5
[2,]    0    5
```

1.3 Network data in R

Lets return to the example from above containing 12 individuals of varying age and sex. It is often worth remembering that writing code in R is highly logical, so it often helps to write out each different step that needs to be done before starting (sometimes this is known as pseudocode). Below, we will: 1. Define an empty matrix with a row for each individual (N = 12) and a column for each possible associate (an NxN adjacency matrix). 2. We will fill in the association data for all the individuals. 3. We will calculate each individual's degree and add this to the data frame we created previously (this example will be a binary network, so the degree will also be binary).

First, we need to create the adjacency matrix. This is an N x N matrix that captures the value of each edge in the social network. It is the fundamental data type in network analysis and is the numeric representation of the social network. If the network is undirected, then the matrix should be symmetrical about the diagonal (top-left to bottom-right). If the matrix is directed, then cells can all contain different values (for example the value in row 5 column 3 does not have to match the value in row 3 column 5). The diagonal represents self-edges that are rarely used in animal networks as these represent individuals interacting with themselves. However, if nodes represent sets of individuals (such as if nodes are species), then it may be meaningful to include information about intra-set interactions. Most network functions in R for performing network measures contain a flag to specify whether the diagonal should be used. Below we provide the code for step 1, continuing in the same session as we defined a population of N=12 (Appendix 1.1).

```
# Define an empty matrix with N rows and N columns
> network <- matrix(0,nrow=N,ncol=N)
```

We can then fill the adjacency matrix with information on whether an individual (the row) associated with each other individuals (the columns). Note that we always keep the diagonal value 0.

```
# Individual 1 (row 1 and all columns). Note that the
# data in c() is in the same order as put into columns
> network[1,] <- c(0,1,0,0,0,0,0,1,0,0,1,0)

# Individual 2. Note that it interacted with individual 1.
> network[2,] <- c(1,0,0,0,0,0,0,0,0,1,0,0)
```

```
# Rest of the individuals
> network[3,] <- c(0,0,0,1,0,0,0,1,0,1,0,0)
> network[4,] <- c(0,0,1,0,0,0,1,0,0,0,0,0)
> network[5,] <- c(0,0,0,0,0,1,0,0,1,0,1,0)
> network[6,] <- c(0,0,0,0,1,0,1,0,0,0,0,1)
> network[7,] <- c(0,0,0,1,0,1,0,0,0,0,0,0)
> network[8,] <- c(1,0,1,0,0,0,0,0,0,0,0,0)
> network[9,] <- c(0,0,0,0,1,0,0,0,0,0,0,0)
> network[10,] <- c(0,1,1,0,0,0,0,0,0,0,0,0)
> network[11,] <- c(1,0,0,0,1,0,0,0,0,0,0,0)
> network[12,] <- c(0,0,0,0,0,1,0,0,0,0,0,0)
```

This results in a square matrix, which is symmetric about the diagonal

```
> network
      [,1] [,2] [,3] [,4] [,5] [,6] [,7] [,8] [,9] [,10] [,11] [,12]
 [1,]    0    1    0    0    0    0    0    1    0     0     1     0
 [2,]    1    0    0    0    0    0    0    0    0     1     0     0
 [3,]    0    0    0    1    0    0    0    1    0     1     0     0
 [4,]    0    0    1    0    0    0    1    0    0     0     0     0
 [5,]    0    0    0    0    0    1    0    0    1     0     1     0
 [6,]    0    0    0    0    1    0    1    0    0     0     0     1
 [7,]    0    0    0    1    0    1    0    0    0     0     0     0
 [8,]    1    0    1    0    0    0    0    0    0     0     0     0
 [9,]    0    0    0    0    1    0    0    0    0     0     0     0
[10,]    0    1    1    0    0    0    0    0    0     0     0     0
[11,]    1    0    0    0    1    0    0    0    0     0     0     0
[12,]    0    0    0    0    0    1    0    0    0     0     0     0
```

We can then calculate each individual's degree (the sum of 1s in each row) to the attribute data we collated in Appendix 1.1 above. Note that new columns can be directly added to data frames.

```
# Add degree of each individual
> attribute_data$Degree <- rowSums(network)
> attribute_data
   ID Age    Sex Degree
1   1   1   Male      3
2   2   1   Male      2
3   3   1 Female      3
4   4   2 Female      2
5   5   3 Female      3
6   6   4   Male      3
7   7   4   Male      2
8   8   4   Male      2
9   9   4 Female      1
10 10   5 Female      2
11 11   5 Female      2
12 12   5   Male      1
```

This code also highlights a useful trick, which is to keep the order of the rows in this data set identical to the row and column ordering of the adjacency matrix in order to facilitate comparison and inclusion of network traits. This also facilitates later analyses as results can be directly added to the attribute data. These data can then be used to investigate if there are any relationships between network position and attributes by adding columns to a regression (which we do in Appendix 2, and also use randomizations to calculate significance).

1.4 Building networks from data in R

Generally, networks are inferred from some observation data. For example, a network may define edges as the probability of associating, which is best calculated automatically rather than doing it manually for each cell in the network. There are two main data structures that contain the data from which networks are typically constructed: the group by individual matrix or the sampling periods matrix. These two formats represent slightly different ways of capturing the data from each observation and facilitate performing null models. My R package *asnipe* provides the functionality to import various data forms into either a group by individual or sampling period matrix, perform randomisations, and generate the adjacency matrix from these. Below we give a brief overview of the structure for each of these matrices.

The group by individual matrix contains a column for each individual in the population, and rows that describe membership to a distinct group. This format is typically used when making observations using the gambit of the group, but can also be used to represent any dyadic data (for example interaction data would contain only two 1s per row). In the case below, 6 individuals were observed forming 6 independent groups, with varying membership.

	Ind."A"	Ind. "B"	Ind. "C"	Ind. "D"	Ind. "E"	Ind. "F"
Group 1	1	0	1	0	0	0
Group 2	0	0	1	1	0	1
Group 3	0	1	0	0	1	0
Group 4	0	0	1	0	0	0
Group 5	0	1	0	0	1	0
Group 6	1	0	0	1	0	1

Sampling periods are a t x N x N matrix, where t is the number of sampling periods, and N is the number of individuals. Here, each N x N sub matrix contains 1s and 0s to represent individuals that were interacted or not during that sampling period. In the case below, all individuals were seen together during the first sampling period, whereas individuals "A" and "B" were not seen together in the second. This approach is most useful when data is captured using stratified sampling, such as focal follows. In this case, a sub matrix represents the observations associated with a round of sampling (or any other time periods when data can be assumed to be independent, such as days). One consideration when using sampling periods is that these can generate very large variables (an extra dimension compared to the group by individual matrix), which could lead to memory usage limitations.

Period 1

	Ind."A"	Ind. "B"	Ind. "C"
Ind."A"	0	1	1
Ind."B"	1	0	1
Ind."C"	1	1	0

Period 2

	Ind."A"	Ind. "B"	Ind. "C"
Ind."A"	0	0	1
Ind."B"	0	0	1
Ind."C"	1	1	0

Period 3

	Ind."A"	Ind. "B"	Ind. "C"
Ind."A"	0	1	0
Ind."B"	1	0	1
Ind."C"	0	1	0

Either of these variables containing the observation data can then be used to generate an adjacency matrix (see Appendix 1.3). Below is an example of a weighted and directed network, where edges represent the probability of an individual (row) being observed following another individual (columns). In the following examples we will be using weighted networks such as this one, as these are the most commonly used in studies of animal social networks.

	Ind."A"	Ind. "B"	Ind. "C"	Ind. "D"	Ind. "E"	Ind. "F"
Ind."A"	0	0.1	0.4	0	0	0.2
Ind."B"	0	0	0.7	0.9	0	0.3
Ind."C"	0	0.5	0	0	0.4	0
Ind."D"	0	0	0.5	0	0	0
Ind."E"	0	0.1	0	0	0	0
Ind."F"	0.3	0	0	0.1	0	0

1.5 Getting network data into R from files

In most cases, matrices will be read in from file rather than being entered manually. The functions read.csv and read.table are very useful here. These generate data frames by default, so if your data is a matrix of numbers, then you can surround the read.csv with as.matrix:

```
> my_network <- as.matrix(read.csv("..../network.csv", header=FALSE,
        row.names=0, stringsAsFactors=FALSE))
> my_attributes <- read.csv("..../attributes.csv", header=TRUE,
        row.names=FALSE)
```

Once a variable has been read from the file into R, it is always useful to check that the first row and the first column actually contain data rather than column names or row names. This can be done either by typing my_network[1,] and my_network[,1] (to check the first row and column separately), or head(my_attributes) to show the first few rows of the data.

Of course, one of the major challenges when starting out with R to work on network data will be to create the grouping data matrices in the first place. In my *asnipe* package (see below), we provide some functions that facilitate this process a bit (it will read in data on groups or group membership in a few different formats and convert these to group by individual or sampling period matrices). However, sometimes raw data does not actually contain the classification of individuals to groups. In these cases there are really few automated tools to help do this because every data file is going to be different. The most efficient way of doing this is to write R code to automate these conversions by using loops and tests (e.g. IF statements, examples of both are contained within the code of the following section) to check if individuals are in the same group and then assign the individuals to groups. For example, if using GPS data, then the best way is to loop through each time point and determine which individuals are within a specified proximity threshold (but note that we generally recommend using clustering algorithms for GPS data, see main text), and to create a new row in the grouping matrix to capture the association between each pair or group of individuals.

Appendix 2: Performing basic network analyses

2.1 Loading up a sample network

Throughout the examples below, we am going to use the sample network contained in my R package *asnipe*, which is available for download from my website (where there are instructions on how to install it). Once installed, the package and example data can be loaded. These data are contained in three variables, a group by individual matrix (347 groups with 151 individuals), information/attributes for each individual (ring number, species and sex for each of 151 individuals), and a one-dimensional matrix containing the time of observation for each group. These can all be accessed as follows:

```
> library(asnipe)
> data("group_by_individual")
> data("individuals")
> data("times")
```

We will use this data to (i) determine whether the adjacency matrix is correlated to a matrix of conspecific identity, (ii) test whether males and females differ in their network degree (both weighted and binary), (iii) investigate the relationship between these, (iv) run an analysis to identify communities, and (v) plot these together with the network. However, the first step after loading a dataset is to check for any inconsistencies (in this case, that the attributes are in a data frame and the number of individuals is consistent). Note that variables for these dataset have been shortened (to gbi, inds and times) when they were included in the package to make it easier to type them out.

```
# Look at the structure of the group by individual variable
> str(gbi)
 int [1:347, 1:151] 0 0 0 0 0 0 0 0 0 0 ...
 - attr(*, "dimnames")=List of 2
  ..$ : chr [1:347] "V1" "V2" "V3" "V4" ...
  ..$ : NULL

# Look at the structure of the attributes variable
> str(inds)
'data.frame':	151 obs. of  4 variables:
```

```
 $ TAG        : Factor w/ 151 levels "0416ECFF87","0416ED0104",..: 111 25 110 ...
 $ RING.NUMBER: Factor w/ 151 levels "C695905","F464557",..: 1 2 3 4 5 6 7 8 9 ...
 $ SPECIES    : Factor w/ 6 levels "BLUTI","COATI",..: 1 4 3 1 3 4 1 1 2 1 ...
 $ SEX        : Factor w/ 3 levels "","F","M": 3 2 2 3 3 3 2 2 3 2 ...

# Look at the structure of the group times variable
> str(times)
 Named num [1:347] 0 22510 24387 15366 21228 ...
 - attr(*, "names")= chr [1:347] "V1" "V2" "V3" "V4" ...
```

These variables all have the same ordering, where the rows of inds correspond to the columns of gbi, and the elements in times to the rows of gbi. The data consists of 151 individuals that were identified in 347 groups. First, we will fix the column names in the group by individual matrix with the ring number, which can be done with a direct assignment:

```
> colnames(gbi) <- inds$RING.NUMBER

# Look at the top few rows and first few columns of gbi
> head(gbi[,1:6])
   C695905 F464557 H300253 H300283 H300296 H839876
V1       0       0       0       0       0       0
V2       0       0       0       0       0       0
V3       0       0       0       0       0       0
V4       0       0       0       0       0       0
V5       0       0       0       0       0       0
V6       0       0       0       0       0       0
```

This shows the first few rows for gbi (and we manually limited this to columns 1:6). Note that because the inds variable is a data frame, the columns can also be directly accessed by name (note that these are case sensitive). In the code above, the value in each row of inds$RING.NUMBER is assigned to each column name in gbi (remember that we specifically maintained the same ordering of individuals in the two variables when we created the data). One of the strengths of using R to perform network analysis is that it provides an environment

in which the results can be directly used in a vast array of statistical tests.

The first step for calculating network statistics is to create an adjacency matrix. This can be done by using the get_network function in my *asnipe* package on either a group by individual matrix or a sampling period matrix.

```
> network <- get_network(gbi)
Generating  151  x  151  matrix
```

In this case, we just use all of the default options, but you may want to check the options for different formats, association indices, and subsetting options in the help file.

We then need to create a matrix that contains the species similarity or difference between individuals in the network (where dyads are assigned 1 if they are the same species, 0 if they are different species). We use this example so that we can provide an overview of two key programming functions that are imperative for network analysis. The most important function to learn when programming is the *for* loop (note that advanced users will prefer to use *apply* functions as these are more efficient, but we present loops here for clarity). In a for loop, the exact same code is re-run, but with a different value for an index.

```
# Use the number of rows in the attributes data as the number of individuals
> N <- nrow(inds)
> N
[1] 151

# Create an empty N x N matrix to store species similarities
> species_sim <- matrix(0, nrow=N, ncol=N)

# Loop through each row and each column in the data
> for (row in c(1:N)) {
>         for (col in c(1:N)) {
>                 # Test if the species are the same
>                 if (inds$SPECIES[row] == inds$SPECIES[col]) {
>                         species_sim[row,col] <- 1
>                 } else {
>                         species_sim[row,col] <- 0
```

```
>                 }
>         }
>}
```

In the code above, we created two loops one inside the other. The result of this is that in the first loop, the variable called row sequentially took the values of 1 throughout to N. For every one of the values of row, a variable called col also sequentially increased from 1 to N. Then for every value of row and col, we tested whether the species associated with the row number was the same as the species associated with the column number (taking the species identities from the inds data frame). The *if* statement is a useful programming structure that performs these logical tests. In this case, if the two species were equal (note that this is tested with a double equal sign), then the code ran what was inside the first set of squiggly brackets (assigning a 1 to that cell/dyad). If they were not equal, then the code in the second set of squiggly brackets was run. The overall result is that we tested the species of each row (from 1 to 151) with each column (from 1 to 151) to create a matrix of species similarity. In the resulting matrix, you can note that the diagonal values are all equal to 1. Remember when using loops and if statements that these need to be opened and closed by squiggly brackets in order to represent the extent of the code to be run.

We can then test whether there is a correlation between the presence of an edge and whether individuals are from the same species. To do this, we first create need to create a binary version of the network, and then conduct a mantel test on the two matrices.

```
# First copy the network into a new variable
> network_binary <- network

# Create a binary network with the following trick
> network_binary[network_binary > 0] <- 1

# Load the mantel test and run it (from Vegan)
> library(vegan)
> mantel(network_binary,species_sim)

Mantel statistic based on Pearson's product-moment correlation
```

```
Call:
mantel(xdis = network_binary, ydis = species_sim)

Mantel statistic r: -0.01252
      Significance: 0.743

Upper quantiles of permutations (null model):
   90\%    95\%  97.5\%    99\%
0.0248 0.0321 0.0394 0.0471

Based on 999 permutations
```

This result suggests that there is no clear relationship between the presence of an edge and species similarity or dissimilarity.

With the same network, we can then calculate network measures in order to determine whether males and females differed in their degree. There are usually many ways of calculating network measures, so below we give several examples for both weighted and binary degree:

```
# Manually measure weighted degree
> deg <- rowSums(network)

# Measure binary degree by creating a temp binary network and
# assign each non-zero value in that network to 1.
# Note that logical tests can be performed inside square brackets
> network_binary <- network
> network_binary[network_binary > 0] <- 1
> deg_binary <- rowSums(network_binary)

# Measure weighted and binary degrees using sna library
# note this package must be manually installed first and
# we detach it after use in order to prevent issues with igraph
# that are caused by both having the same function names.
```

```
> library(sna)
> deg <- degree(network, gmode="graph")
> deg_binary <- degree(network, gmode="graph",ignore.eval=TRUE)
> detach("package:sna")

# Measure weighted and binary degrees using graph library
# note this package must be manually installed first
# and network has to be converted to a graph variable
> library(igraph)
> net_graph <- graph.adjacency(network,mode="undirected",weighted=TRUE,diag=FALSE)
> deg <- graph.strength(net_graph)
> deg_binary <- degree(net_graph)
> detach("package:igraph")

# Add degrees to attributes data
> inds$DEGREE <- deg
> inds$DEGREE_BINARY <- deg_binary

# Plot the difference in degrees by sex, and their relationship on a 3 panel plot:

# First fix empty sexes by setting them to NA which R can deal with easily
> inds$SEX[inds$SEX == ""] <- NA

# Then drop empty factors (to avoid plotting values without a sex)
> inds$SEX <- factor(inds$SEX)

# Finally set up a 3 panel figure and plot each relationship ignoring NAs
> par(mfrow=c(1,3))
> boxplot(DEGREE~SEX,data=na.omit(inds), col=c("red","blue"))
> boxplot(DEGREE_BINARY~SEX,data=na.omit(inds), col=c("red","blue"))
> plot(DEGREE~DEGREE_BINARY,data=na.omit(inds), col=c("red","blue")[SEX])
> legend("bottomright",c("Female","Male"),col=c("red","blue"),pch=1)
```

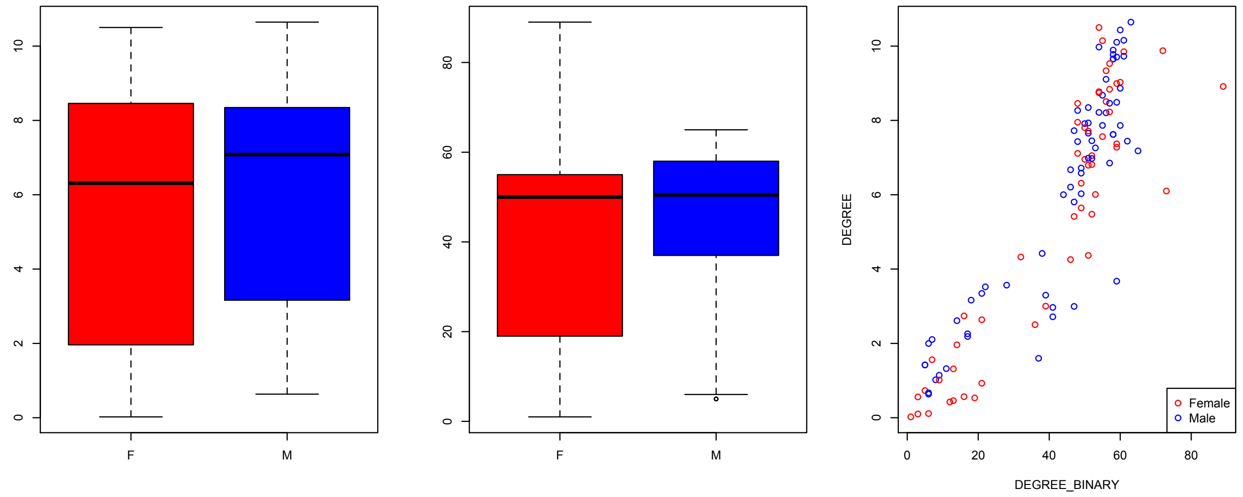

Figure 1: Male and female weighted degree, binary degree, and the relationship between these.

We can also calculate the mean (non-zero) edge weights of male and female edges from the adjacency matrix:

```
# Select all rows and all columns of the network where individual is male
> network_male <- network[which(inds$SEX=="M"), which(inds$SEX=="M")]
> mean(network_male[network_male>0])
[1] 0.1481028

# Select all rows and all columns of the network where individual is female
> network_female <- network[which(inds$SEX=="F"), which(inds$SEX=="F")]
> mean(network_female[network_female>0])
[1] 0.1281252
```

To test if there is a significant difference between males and females, we can either use data permutations and node permutations, and compare the test statistic from the observed to 1000 randomised t-statistics. Note that there will often be a difference in the P value between the t-test and the permutation, highlighting how anti-conservative parametric statistics are due to over-inflated degrees of freedom.

```
# Calculate the observed statistic
> network_male <- network[which(inds$SEX=="M"), which(inds$SEX=="M")]
> network_female <- network[which(inds$SEX=="F"), which(inds$SEX=="F")]
```

```
> t.test(network_female[network_female>0], network_male[network_male>0])

        Welch Two Sample t-test

data:  network_female[network_female > 0] and network_male[network_male > 0]
t = -4.9374, df = 1518.947, p-value = 8.791e-07
alternative hypothesis: true difference in means is not equal to 0
95 percent confidence interval:
 -0.02791431 -0.01204090
sample estimates:
mean of x mean of y
0.1281252 0.1481028

# Store the test statistic (t-value) from the observed dataW
> t_obs <- t.test(network_female[network_female>0], network_male[network_male>0])$statistic
> t_obs
       t
-4.93739

# Get 1000 networks from data-stream permutation
> random_networks <- network_permutation(gbi,association_matrix=network, permutations=1000)
Starting permutations, generating  1000  x  151  x  151  matrix

# Create a 1D matrix to store results
> t_rand_DS <- rep(0,1000)

# Get test statistic from the 1000 random networks
# note that i represents the index of random networks (from 1 to 1000)
# and is also used in the results matrix where we store the value of the t test
> for (i in c(1:1000)) {
>        net_male_rand <- random_networks[i,which(inds$SEX=="M"), which(inds$SEX=="M")]
>        net_female_rand <- random_networks[i,which(inds$SEX=="F"), which(inds$SEX=="F")]
>        t_rand_DS[i] <- t.test(net_female_rand[net_female_rand>0],
```

```
                net_male_rand[net_male_rand>0])$statistic
> }

# Get test statistic from 1000 node permutations
# using rmperm from SNA library
# Create a 1D matrix to store results
> library(sna)
> t_rand_NP <- rep(0,1000)
> for (i in c(1:1000)) {
>        random_network <- rmperm(network)
>       net_male_rand <- random_network[which(inds$SEX=="M"), which(inds$SEX=="M")]
>       net_female_rand <- random_network[which(inds$SEX=="F"), which(inds$SEX=="F")]
>       t_rand_NP[i] <- t.test(net_female_rand[net_female_rand>0],
                net_male_rand[net_male_rand>0])$statistic
> }

# Detach the package sna in order to avoid issues with igraph
> detach("package:sna")

# Plot observed versus posterior distributions
> par(mfrow=c(1,2))
> hist(t_rand_DS,breaks=100)
> abline(v=t_obs, col="red")
> hist(t_rand_NP, breaks=100)
> abline(v=t_obs, col="red")
```

These plots show where the t-value of the observed difference between males and females (red lines) sits compared to the distribution of t-values from the randomised data (Figure 2). Randomisations can then be used to directly calculate the P value by comparing the observed test statistic (in this case the t-value) to the test statistic derived from randomised data. When the difference is significant, the observed value will sit outside of the random, or null, distribution. The significance of the result is therefore calculated by counting the number of randomised test statistics that had a greater (when the statistic is positive) or lower (when it is negative negative) value than the observed, and dividing this value by the number of randomisations (i.e. the probability that a random

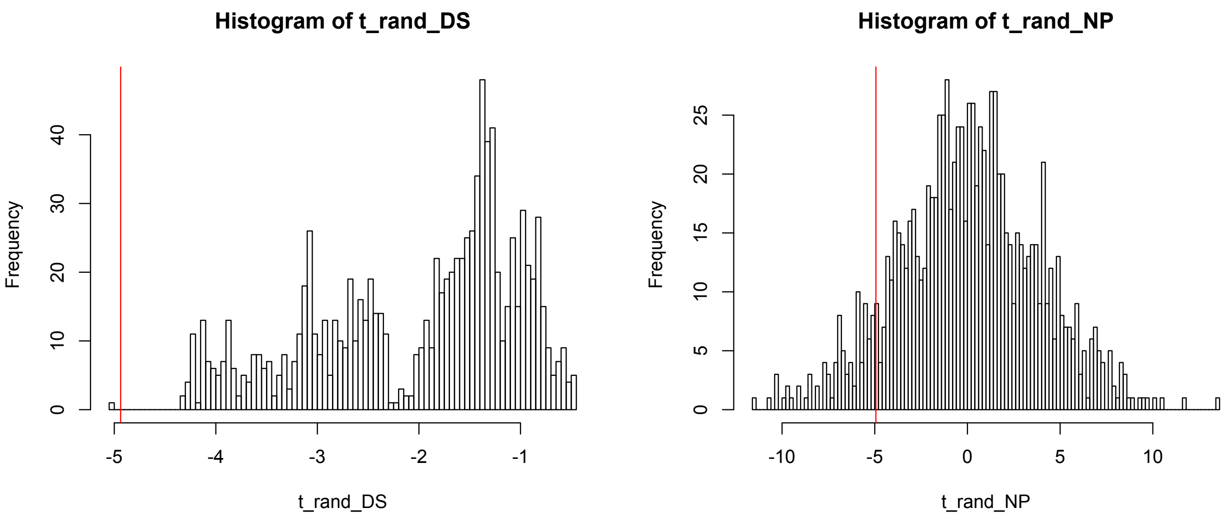

Figure 2: Histogram comparing the t-values from observed data (red line) to t-statistics calculated on randomised networks based permutation of the data stream (left) and of the node labels (right). Because node-label permutations make major changes to the relationship between node attributes and network position, they can create disproportionately broad random distributions (right) compared to data-based permutations (left). This can result in a higher rate of false negatives.

network would be more different than the observed).

```
# Get a P value (remember to divide by the number of permutations)
> sum(abs(t_obs) < abs(t_rand_DS))/1000
[1] 0.016

> sum(abs(t_obs) < abs(t_rand_NP))/1000
[1] 0.169
```

Finally, we want to visually determine how individuals are connected by running a community analysis and plotting the network. It is useful to visualise species identity of each node by giving a different colour to each species. Community membership can be determined and visualized by plotting the network in igraph. Note that the plotting function requires a list of community members in order to draw the polygons. Lists are a type of variable that can store different types of data (or matrices of different sizes) in each element (i.e. cell). Cells in a list are typically accessed using double square brackets.

```
# Load igraph
> library(igraph)
```

```
# Convert adjacency matrix to igraph variable
> net_graph <- graph.adjacency(network,mode="undirected",weighted=TRUE,diag=FALSE)

# Infer what community each node is in
> community <- leading.eigenvector.community(net_graph)

# Convert to a list (as required by the plotting function)
> communities <- list()
> for (i in 1:max(community$membership)) {
>        communities[[i]] <- which(community$membership == i)
> }

# Define a colour for each species
> cols <- c("lightblue", "steelblue", "gold", "honeydew4", "brown2")

# Plot the results
> plot(net_graph, vertex.color=cols[inds$SPECIES], vertex.size=3, vertex.label=NA,
        + mark.groups=communities)
```

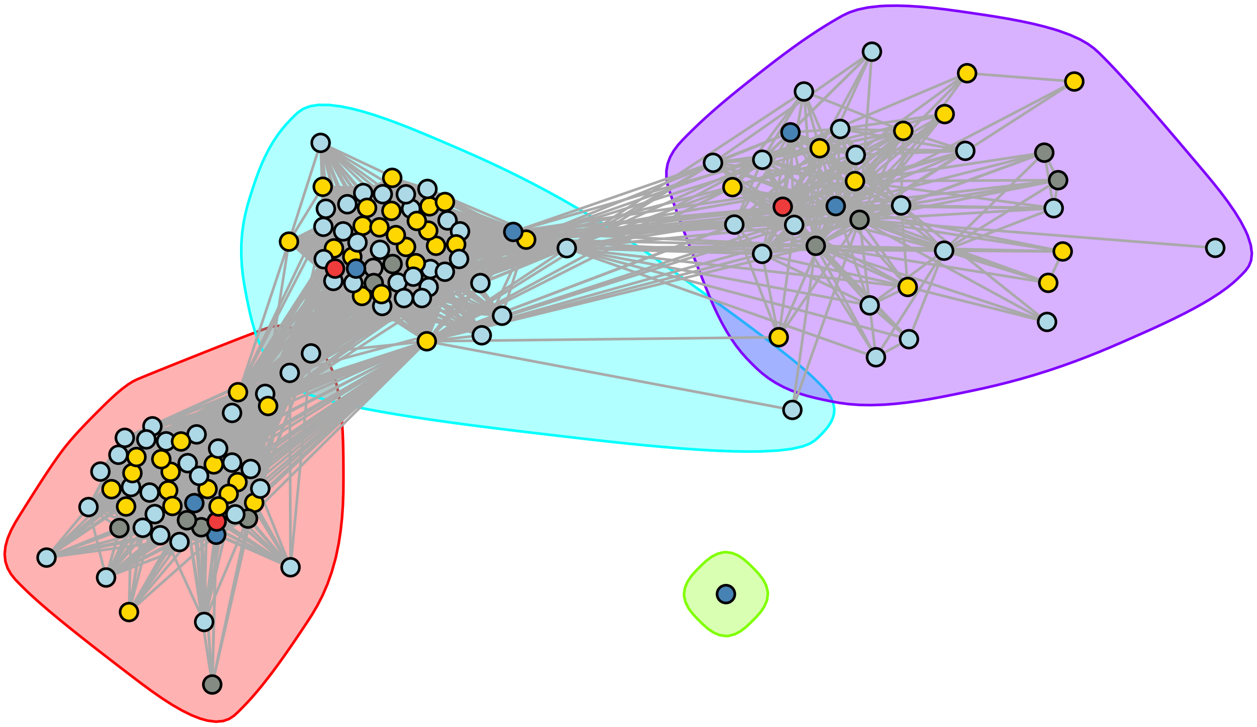

Figure 3: Example of a plotted network, highlighting the community detections and the relative position of individuals in the social network. Here, differently coloured nodes represent species, and communities are highlighted using background colours. The single point is an isolate (a node with no connections), and sits in it's own community.

The process in this appendix can be repeated for almost any possible network measure (see the help files for the sna and igraph packages). However, often we also need to describe more fundamental properties of networks. We give some examples of how these things can be done below:

Calculate the number of edges in a directed network

```
> sum(network>0)
[1] 6274
```

Calculate the number of edges in an undirected network

```
> sum(network>0)/2
[1] 3137
```

Calculate the mean number of associates (binary network)

```
> network_binary <- network
> network_binary[network_binary > 0] <- 1
> mean(rowSums(network_binary))
[1] 41.54967
```

Calculate the mean (non-zero) edge weight

```
> mean(network[network>0])
[1] 0.1351232
```

Appendix 3: Simulation code for Box 4

```
# Load required libraries
library(igraph)
library(sna)
library(asnipe)
library(lme4)

# Set parameters
areas <- 2
N <- 20
sampling_periods <- 100

# Generate nodes
ids <- data.frame(ID=1:(areas*N),AREA=rep(1:areas,each=N),DEG_DIST=NA)

# Generate a degree distribution and normalise it
for (i in 1:areas) {
        degree_distribution <- rpois(N,3)+i
        ids$DEG_DIST[ids$AREA==i] <- degree_distribution/max(degree_distribution)
}

# Generate attributes
# This creates a correlation between sex and underlying degree probability
ids$SEX <- sapply(ids$DEG_DIST,FUN=function(x) { sample(c("M","F"),1,prob=c(x,1-x))})
#ids$SEX <- sample(c("M","F"),N*areas,replace=TRUE)

# Generate probability for each edge (only within area)
probs <- outer(ids$DEG_DIST, ids$DEG_DIST, "*")*outer(ids$AREA,ids$AREA,"==")

# Create sampling periods, each sample only contains data from one area
sps <- array(0,c(areas*sampling_periods,N*areas,N*areas))
```

```
for (i in 1:areas) {
        sps[((sampling_periods*(i-1))+1):(sampling_periods*i),
                ((N*(i-1))+1):(N*i),((N*(i-1))+1):(N*i)]
                <- rgraph(N,m=sampling_periods,tprob=probs[((N*(i-1))+1):(N*i),
                ((N*(i-1))+1):(N*i)],mode="graph")
}

# Generate network
network <- get_network(sps,data_format="SP")
rownames(network) <- ids$SEX
colnames(network) <- ids$SEX

# Calculate degrees
ids$DEGREE <- degree(network,gmode="graph")

# Calculate effect
coef <- fixef(lmer(DEGREE~SEX+(1|AREA),data=ids))[2]

# Create random networks, randomising within day and within area
networks_rand <- network_permutation(sps,data_format="SP",
        association_matrix=network,days=rep(c(1:sampling_periods),2),
        within_day=TRUE,locations=rep(1:areas,each=sampling_periods),within_location=TRUE)

# Calculate degree distribution for each network
deg_rand <- apply(networks_rand,1,function(x) { degree(x,gmode="graph")})

# Get coefficients for each randomisation
coefs <- apply(deg_rand,2,function(x) { fixef(lmer(x~SEX+(1|AREA),data=ids))[2] })


# Plot results
par(mfrow=c(1,3),cex.lab=1.5)
```

```
# Plot network
plot(graph.adjacency(network,mode="undirected",diag=FALSE,
        weighted=TRUE, add.rownames="code"),vertex.size=ids$DEGREE*3)

# Plot observed difference
plot(ids$DEGREE~factor(ids$SEX),xlab="Sex",
        ylab="Strength (weighted degree)",ylim=c(0,max(ids$DEGREE)))

# Plot resulting distribution
a <- hist(coefs,xlim=c(min(coefs),coef),col="black",main="",
        xlab="Coefficient value",ylab="Frequency",breaks=100)
segments(coef,0,coef,max(a$counts),col="red")

# Model results
summary(lmer(DEGREE~SEX+(1|AREA),data=ids))
```

Appendix 4: Simulation code for Box 5

```
# Load required libraries

library(igraph)
library(sna)
library(asnipe)
library(lme4)

# Set parameters
areas <- 2
N <- 20
sampling_periods <- 100

# Generate nodes
ids <- data.frame(ID=1:(areas*N),AREA=rep(1:areas,each=N),DEG_DIST=NA)

# Generate a degree distribution and normalise it
for (i in 1:areas) {
        degree_distribution <- rpois(N,3)+i
        ids$DEG_DIST[ids$AREA==i] <- degree_distribution/max(degree_distribution)
}

# Generate attributes
# This creates a correlation between sex and underlying degree probability
ids$SEX <- sample(c("M","F"),N*areas,replace=TRUE)

# Generate probability of being observed (males=1,females=2)
ids$OBS_PROB <- 0.7
ids$OBS_PROB[which(ids$SEX=="M")] <- 1

# Generate probability for each edge (only within area)
```

```
probs <- outer(ids$DEG_DIST, ids$DEG_DIST, "*")*outer(ids$AREA,ids$AREA,"==")

# Create sampling periods, each sample only contains data from one area
sps <- array(0,c(areas*sampling_periods,N*areas,N*areas))
for (i in 1:areas) {
        sps[((sampling_periods*(i-1))+1):(sampling_periods*i),
                ((N*(i-1))+1):(N*i),((N*(i-1))+1):(N*i)]
                <- rgraph(N,m=sampling_periods,
                tprob=probs[((N*(i-1))+1):(N*i),((N*(i-1))+1):(N*i)],mode="graph")
}

# Remove data when individuals were not observed
sps2 <- sps
for (i in 1:(N*areas)) {
        s <- sample(c(0,1),200,replace=TRUE,prob=c(1-ids$OBS_PROB[i],ids$OBS_PROB[i]))
        sps2[which(s==0),i,] <- 0
        sps2[which(s==0),,i] <- 0
}

# Generate original network
network_original <- get_network(sps,data_format="SP")
rownames(network_original) <- ids$SEX
colnames(network_original) <- ids$SEX

# Generate subsampled network
network <- get_network(sps2,data_format="SP")
rownames(network) <- ids$SEX
colnames(network) <- ids$SEX

# Calculate degrees
ids$DEGREE_ORIG <- degree(network_original,gmode="graph")
ids$DEGREE <- degree(network,gmode="graph")
```

```
# Calculate effect
coef <- fixef(lmer(DEGREE~SEX+(1|AREA),data=ids))[2]

# Create random networks, randomising within day and within area
networks_rand <- network_permutation(sps2,data_format="SP",
        association_matrix=network,days=rep(c(1:sampling_periods),2),
        within_day=TRUE,locations=rep(1:areas,each=sampling_periods),within_location=TRUE)

# Calculate degree distribution for each network
deg_rand <- apply(networks_rand,1,function(x) { degree(x,gmode="graph")})

# Get coefficients for each randomisation
coefs <- apply(deg_rand,2,function(x) { fixef(lmer(x~SEX+(1|AREA),data=ids))[2] })

# Plot results
par(mfrow=c(1,3),cex.lab=1.5)

# Plot network
plot(ids$DEGREE_ORIG~factor(ids$SEX),xlab="Sex",
        ylab="Strength (weighted degree)",ylim=c(0,max(ids$DEGREE_ORIG)))

# Plot observed difference
plot(ids$DEGREE~factor(ids$SEX),xlab="Sex",
        ylab="Strength (weighted degree)",ylim=c(0,max(ids$DEGREE)))

# Plot resulting distribution
a <- hist(coefs,xlim=c(min(coefs),max(coefs)),col="black",
        main="",xlab="Coefficient value",ylab="Frequency")
segments(coef,0,coef,max(a$counts),col="red")

# Model results
summary(lmer(DEGREE~SEX+(1|AREA),data=ids))
```
